# Tick diversity and molecular detection of *Anaplasma*, *Babesia*, and *Theileria* from Khao Kheow open zoo, Chonburi Province, Thailand

**DOI:** 10.3389/fvets.2024.1430892

**Published:** 2024-07-01

**Authors:** Chalida Sri-in, Kritsada Thongmeesee, Wittawat Wechtaisong, Nichapat Yurayart, Ganyawee Rittisornthanoo, Chatlada Akarapas, Natcha Bunphungbaramee, Natthanicha Sipraya, Elizabeth Riana, Thuong Thi Huyen Bui, Patchana Kamkong, Umaporn Maikaew, Piyaporn Kongmakee, Arpussara Saedan, Lyric C. Bartholomay, Sonthaya Tiawsirisup

**Affiliations:** ^1^Center of Excellence in Animal Vector-Borne Diseases, Veterinary Parasitology Unit, Department of Veterinary Pathology, Faculty of Veterinary Science, Chulalongkorn University, Bangkok, Thailand; ^2^Veterinary Pathobiology Graduate Program, Faculty of Veterinary Science, Chulalongkorn University, Bangkok, Thailand; ^3^6^th^ Year Veterinary Student, Academic Year 2022, Faculty of Veterinary Science, Chulalongkorn University, Bangkok, Thailand; ^4^International Graduate Program of Veterinary Science and Technology, Faculty of Veterinary Science, Chulalongkorn University, Bangkok, Thailand; ^5^Parasitology Unit, Department of Pathology, Faculty of Veterinary Science, Chulalongkorn University, Bangkok, Thailand; ^6^Khao Kheow Open Zoo, Zoological Park Organization, Bangpra, Sriracha, Chonburi, Thailand; ^7^Animal Conservation and Research Institute, The Zoological Park Organization of Thailand, Bangkok, Thailand; ^8^Department of Pathobiological Sciences, School of Veterinary Medicine, University of Wisconsin-Madison, Madison, WI, United States

**Keywords:** *Anaplasma*, *Babesia*, *Theileria*, Thailand, tick, zoo wildlife

## Abstract

Ticks are obligate blood-feeding ectoparasites notorious for their role as vectors for various pathogens, posing health risks to pets, livestock, wildlife, and humans. Wildlife also notably serves as reservoir hosts for tick-borne pathogens and plays a pivotal role in the maintenance and dissemination of these pathogenic agents within ecosystems. This study investigated the diversity of ticks and pathogens in wildlife and their habitat by examining ticks collected at Khao Kheow Open Zoo, Chonburi Province, Thailand. Tick samples were collected for 1 year from March 2021 to March 2022 by vegetation dragging and direct sampling from wildlife. A total of 10,436 ticks or 449 tick pools (1–50 ticks per pool) underwent screening for pathogen presence through conventional PCR and DNA sequencing. Out of the 298 samples (66.37%) where bacteria and protozoa were detected, encompassing 8,144 ticks at all stages, 114 positive samples from the PCR screenings were specifically chosen for detailed nucleotide sequencing and comprehensive analysis. Four species of ticks were conclusively identified through the application of PCR, namely, *Rhipicephalus microplus*, *Dermacentor auratus, Haemaphysalis lagrangei*, and *Haemaphysalis wellingtoni*. The highest infection rate recorded was for *Anaplasma* spp. at 55.23% (248/449), followed by *Babesia* spp. and *Theileria* spp. at 29.62% (133/449) and 16.26% (73/449), respectively. Among bacteria identified, three *Anaplasma* genotypes were closely related to an unidentified *Anaplasma* spp., *A. phagocytophilum*, and *A. bovis*. Among protozoa, only an unidentified *Babesia* spp. was found, whereas two *Theileria* genotypes found were closely related to unidentified *Theileria* spp. and *T. equi*. Significantly, our findings revealed coinfection with *Anaplasma* spp., *Theileria* spp., and *Babesia* spp. While blood samples from wildlife were not specifically collected to assess infection in this study, the data on the presence of various pathogens in ticks observed can serve as valuable indicators to assess the health status of wildlife populations and to monitor disease dynamics. The findings could be valuable in developing programs for the treatment, prevention, and control of tick-borne illnesses in this area. However, additional research is required to determine the ticks’ ability to transmit these pathogens and enhance the current understanding of the relationship among pathogens, ticks, and hosts.

## 1 Introduction

Ticks belong to the class Arachnida, subclass Acari, and are classified into three families: (i) Ixodidae (hard ticks); (ii) Argasidae (soft ticks); and (iii) Nuttalliellidae. There are approximately 899 hard tick species, and 185 soft tick species are known (1), some of which act as vectors for a broad range of pathogens in domestic animals, wildlife, and humans. These pathogens affect the health of animals and humans, consequently causing significant economic losses worldwide (2). Additionally, to this consequence, tick bites may result in paralysis and toxicoses (3). Furthermore, ticks of all developmental stages can induce allergic reactions in the host’s skin (4). Their feeding behavior also makes ticks responsible for direct skin damage to the host (2).

In humans, tick-borne infectious diseases include babesiosis, caused by the protozoa *Babesia microti* (5); Lyme disease, caused by *Borrelia burgdorferi* (6); and human granulocytic anaplasmosis, caused by *Anaplasma phagocytophilum* (7). In animals, tick-borne infectious diseases include Crimean–Congo hemorrhagic fever and tick-borne encephalitis virus caused by viruses; Q fever, borreliosis, and relapsing fever, anaplasmosis, and ehrlichiosis caused by bacteria; and theileriosis and babesiosis caused by protozoa (8).

Wildlife is considered an important reservoir of tick-borne protozoal and bacterial pathogens, including *Theileria*, *Babesia,* and *Anaplasma* (9). Wildlife infected by tick-borne pathogens may show only mild symptoms, which makes the diseases difficult to diagnose (10). Thus, these pathogens can circulate among wildlife and tick populations for extended periods before being identified (11). Since disease transmission occurs between wildlife and other hosts via ticks, pathogen identification in ticks can help in disease surveillance. Conventional polymerase chain reaction (PCR) is a comprehensive, specific, and rapid technique for tick species identification and pathogen diagnosis in ticks (12). We performed PCR and DNA sequencing to investigate the diversity of and identify pathogens in tick specimens collected from the various wildlife species at Khao Kheow Open Zoo, Chonburi Province, Thailand.

Khao Kheow Open Zoo is the largest open zoo in Thailand. It covers an area of about 2,000 acres and contains more than 8,000 animals from more than 300 species of wildlife. The zoo is situated within the boundaries of the Khao Kheow Wildlife Sanctuary and was established to rescue injured wildlife and support research about the diversity of the environment and ecosystem. Consequently, certain species of wildlife can move between these two areas and in doing so, transmit pathogens. Given the variety of host species and the possible modes of disease transmission among them, the Khao Kheow Open Zoo is a great resource for studying tick diversity and therefore the possible role of ticks as vectors for important pathogens. In this present study, ticks were collected from tapir, deer, Eld’s deer, spotted deer, barking deer, and hybrid cow, and by vegetation dragging in tapir and Eld’s deer habitat. We offer insights that can inform programs for the prevention and control of ticks and tick-borne diseases in wildlife at the Khao Kheow Open Zoo. Furthermore, the study findings can be applied to reducing disease transmission between zoo wildlife and livestock, thereby reducing the economic loss from such diseases, improving wildlife conservation, and monitoring the progression of tick-borne diseases in Thailand.

## 2 Materials and methods

### 2.1 Tick collection and identification

**Figure 1:**
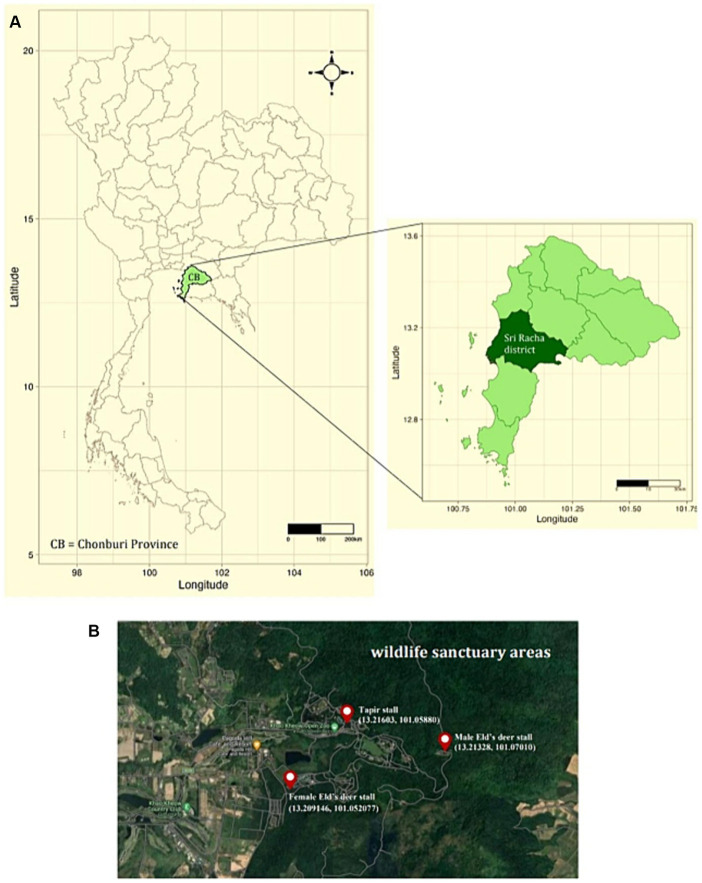
**(A)** Map of Thailand depicting sampling sites in Sri Racha District, Chonburi Province. **(B)** Study sites at Khao Kheow Open Zoo, Chonburi Province of Thailand. The three sampling locations were the tapir, male, and female Eld’s deer habitats.

Two methods were used to collect tick specimens. First, ticks were directly collected from resident wildlife, including tapirs (*Tapirus indicus*), Eld’s deer (*Rucervus eldii*), spotted deer (*Axis axis*), red deer (*Cervus elaphus*), barasingha (*Rucervus duvaucelii*), and hybrid cows—a mix of domestic cattle, red cattle, and bulls (*Bos gaurus hubbacki*) in their lineage—during routine health checks at the zoo following anesthesia. Second, ticks were obtained via vegetation dragging. Researchers randomly traversed animal trails within the Eld’s deer and tapir habitats for 1 h at each site and collected ticks from the vegetation every month. Three tick collection sites were established for vegetation dragging, namely, the female Eld’s deer (13.209146, 101.052077), male Eld’s deer (13.21328, 101.07010), and tapir (13.21603, 101.05880) habitats (Figure 1). All collected tick specimens were stored in microcentrifuge tubes with RNA stabilization solution (RNA Later^™^ Soln, Invitrogen, United States) and transported to the Parasitology Unit, Faculty of Veterinary Science, Chulalongkorn University, Bangkok, Thailand. The samples were stored at −40°C until further examination. Morphological identification was employed to categorize the collected ticks according to morphology, sex, and life stage, following established protocols (13). Subsequently, tick species were identified through the polymerase chain reaction technique and DNA sequencing.

### 2.2 DNA extraction and conventional polymerase chain reaction

Conventional polymerase chain reaction (PCR) was used for tick species identification and pathogen detection. Due to the substantial quantity, the collected larvae and nymphs were subdivided into pools containing 1 to 50 larvae, and 1 to 10 nymphs, categorized based on morphology, location, and collection time (14, 15). For adult ticks, females and males were examined individually. Tick samples, encompassing males, females, nymph pools, and larval pools, underwent screening for pathogen presence, including *Anaplasma, Babesia*, and *Theileria,* utilizing PCR and DNA sequencing techniques (Supplementary Table 1).

For DNA extraction, the tick samples were homogenized, and genomic DNA was extracted using an IndiSpin Pathogen Kit (Indical Bioscience, Germany) according to the manufacturer’s instructions. Nucleic acid samples were stored at −80°C. PCR was performed using KOD One^™^ PCR Master Mix Blue (TOYOBO, Japan). Each 25-μL reaction mixture contained the following components: 10 μM forward primer (0.75 μL), 10 μM reverse primer (0.75 μL), template DNA (1 μL) or distilled water (1 μL; for the negative control), KOD PCR master mix (12.5 μL), and distilled water (10 μL). The PCR products were electrophoresed on a 1.5% agarose gel mixed with RedSafe™ (iNtRON Biotechnology, South Korea), and the expected bands were visualized using a UV transilluminator. The oligonucleotide primers, product sizes, and cycle conditions required to identify tick species and pathogens within ticks are detailed in Table 1.

**Table 1:** Primers used for tick and pathogen identification.

Identification	Target gene	Primer name	Oligonucleotide primer (5′… 3′)	Product size (bp)	References
Tick	*16S rRNA*	16S + 1 16S-1	CTGCTCAATGATTTTTTAAATTGCTGTGG CCGGTCTGAACTCAGATCAAGT	401–460	(16)
*Babesia*	*18S rRNA*	Bab-F Bab-R	GTTTCTGMCCCATCAGCTTGAC CAAGACAAAAGTCTGCTTGAAAC	420–440	(17)
*Theileria*	*18S rRNA*	989-F 990-R	AGTTTCTGACCTATCAG TTGCCTTAAACTTCCTTG	1,098	(18)
Anaplasmataceae Family	*16S rRNA*	EHR16SD EHR16SR	GGTACCYACAGAAGAAGTCC TAGCACTCATCGTTTACAGC	345	(19)

Positive PCR products showing DNA bands of the expected size were cut from the agarose gel and purified using the GenepHlow^™^ Gel/PCR cleanup Kit (Geneaid Biotech, Taiwan) following the manufacturer’s protocols. The PCR products were then submitted to a commercial service, U2Bio (Bangkok, Thailand), for DNA sequencing. The quantity and quality were assessed using the NanoDrop^™^ 1,000 Spectrophotometer, and only samples with Genomic DNA (gDNA) concentration exceeding 30 ng/μL were chosen for sequencing.

### 2.3 DNA sequencing and phylogenetic analysis

The nucleotide sequencing results were aligned and trimmed using the ClustalW multiple alignments tool (20) on the Molecular Evolutionary Genetics Analysis software version 10.0 X (MEGA X) (21) and compared with reference DNA sequences in the GenBank database. BLASTn was used to identify genera and/or species of ticks and pathogens (22). The sequences were subjected to genetic diversity analysis using the DnaSP6 software^1^ to determine haplotype or nucleotide sequence type diversity. The identified haplotypes were uploaded to the GenBank database. To visualize the sequences of different haplotypes, we used Population Analysis with Reticulate Trees version 1.7 (PopART 1.7) (23), a software for population genetics analysis that generates haplotype networks. These networks implement minimum spanning and median-joining network methods (24). For phylogenetic analysis, the optimal model of nucleotide substitution was determined using the Find Best DNA/Protein Model implemented in MEGA X (21). The best-fitted model selected for constructing the phylogenetics of each dataset was the one with the lowest Bayesian Information Criterion score. The phylogenetic of the protozoal, bacterial, and tick DNA sequences identified were generated using the maximum likelihood method based on the best-fitted models using MEGA X (21). The robustness of the phylogenetic was estimated using 1,000 bootstrap replicates.

### 2.4 Statistical analysis

Statistical analysis was performed using the Chi-square test and two-way ANOVA implemented in GraphPad Prism version 9.4.1 (GraphPad Software Inc., La Jolla, CA, United States). Statistical significance was set at *p*-value <0.05.

## 3 Results

### 3.1 Tick collection and identification

A total of 10,436 ticks were collected during the study. The vast majority, comprising 97.26% (10,150 out of 10,436), were obtained through vegetation dragging, whereas the remaining 2.74% (286 out of 10,436) were directly collected from hosts. Among these, most ticks were retrieved from the soft areas of wildlife, notably from regions such as the inguinal area, front and hind legs, ears, and neck (Supplementary Figure 1A). Of the ticks collected, larvae were the most abundant (96.4%, 10,060/10,436), followed by nymphs (1.86%, 194/10,436), adult females (1.24%, 129/10,436), adult males (0.51%, 53/10,436; Supplementary Figure 1B). In total, 449 pool samples were established, of which 48.1% consisted of larvae (216/449), 28.73% of adult females (129/449), 11.8% of adult males (53/449), and 11.36% of nymphs (51/449; Supplementary Figure 1C).

#### 3.1.1 Phylogenetic analysis of collected ticks

NCBI BLASTn was used to generate mitochondrial *16S rRNA* gene sequences from 114 samples (1,338 ticks), all of which contained pathogens. All *16S rRNA* gene sequences generated were aligned using MEGA X, and analyses were conducted using BLASTn and DnaSP6. The outcomes of BLASTn and haplotype analyses are presented in Table 2, which identified 16 haplotypes across three genera and four species: *Rhipicephalus microplus* 35.96% (41/114) was grouped in haplotype 1–2 (OQ918450-51), *Dermacentor auratus* 2.63% (3/114) was grouped in haplotype 3 (OQ918452), *Haemaphysalis* (*H.*) *wellingtoni* 0.88% (1/114) was grouped in haplotype 4 (OQ918453), and *H. lagrangei* 60.53% (69/114) was grouped in haplotype 5–16 (OQ918454-64). These results were used to construct a phylogenetic for comparisons with other ixodid tick species registered on the GenBank database.

**Table 2:** Identification of tick species collected from Khao Kheow Open Zoo, Chonburi Province, Thailand based on nucleotide sequence analysis using the nucleotide basic local alignment search tool.

Haplotype	No. of sequences (*N* = 114)	BLASTn			Length (bps)	Submitted sequences accession number
		Closest sequence	Species	% identity		
1	20	MN650726	*Rhipicephalus microplus*	100	409	OQ918450
2	21	MN650726	*R. microplus*	99.75	409	OQ918451
3	3	MT371592	*Dermacentor auratus*	99.75	409	OQ918452
4	1	MG874023	*Haemaphysalis wellingtoni*	100	409	OQ918453
5	40	MG788690	*H. lagrangei*	100	409	OQ918454
6	4	MZ490779	*H. lagrangei*	99.75	409	OQ918455
7	3	MG788690	*H. lagrangei*	99.75	409	OQ918456
8	2	MG788690	*H. lagrangei*	99.75	409	OQ918457
9	7	MZ490779	*H. lagrangei*	99.75	409	OQ918458
10	3	MG788690	*H. lagrangei*	99.51	409	OQ918459
11	2	MZ490779	*H. lagrangei*	99.51	409	OQ918460
12	2	MZ490779	*H. lagrangei*	99.51	409	OQ918461
13	2	MG788690	*H. lagrangei*	99.51	409	OQ918462
14	2	KC170731	*H. lagrangei*	99.27	409	OQ918463
15	1	MZ490779	*H. lagrangei*	99.27	409	OQ918464
16	1	MG788690	*H. lagrangei*	99.26	409	OQ918465

BLASTn, nucleotide Basic Local Alignment Search Tool.

**Figure 2:**
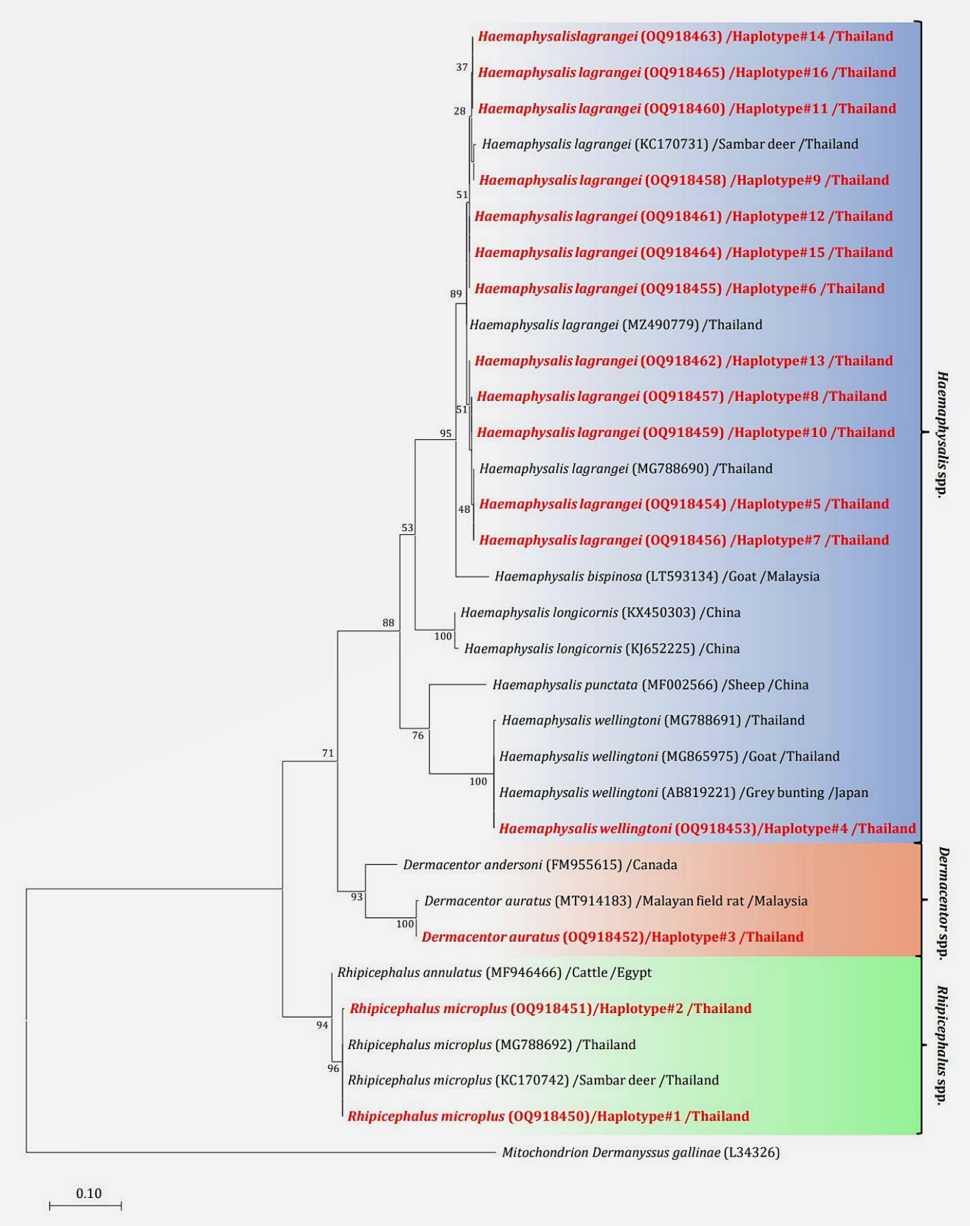
Maximum likelihood phylogenetic tree (413 nucleotide sites) from partial mitochondrial *16S rRNA* genes of the tick species found in this study (highlighted in red) and global isolates. Nucleotide sequences using the Tamura 3-parameter model with 1,000 bootstrap replications and using mitochondria from *Dermanyssus gallinae* as the outgroup. The tree is drawn to scale, with branch lengths indicating the number of substitutions per site.

**Figure 3:**
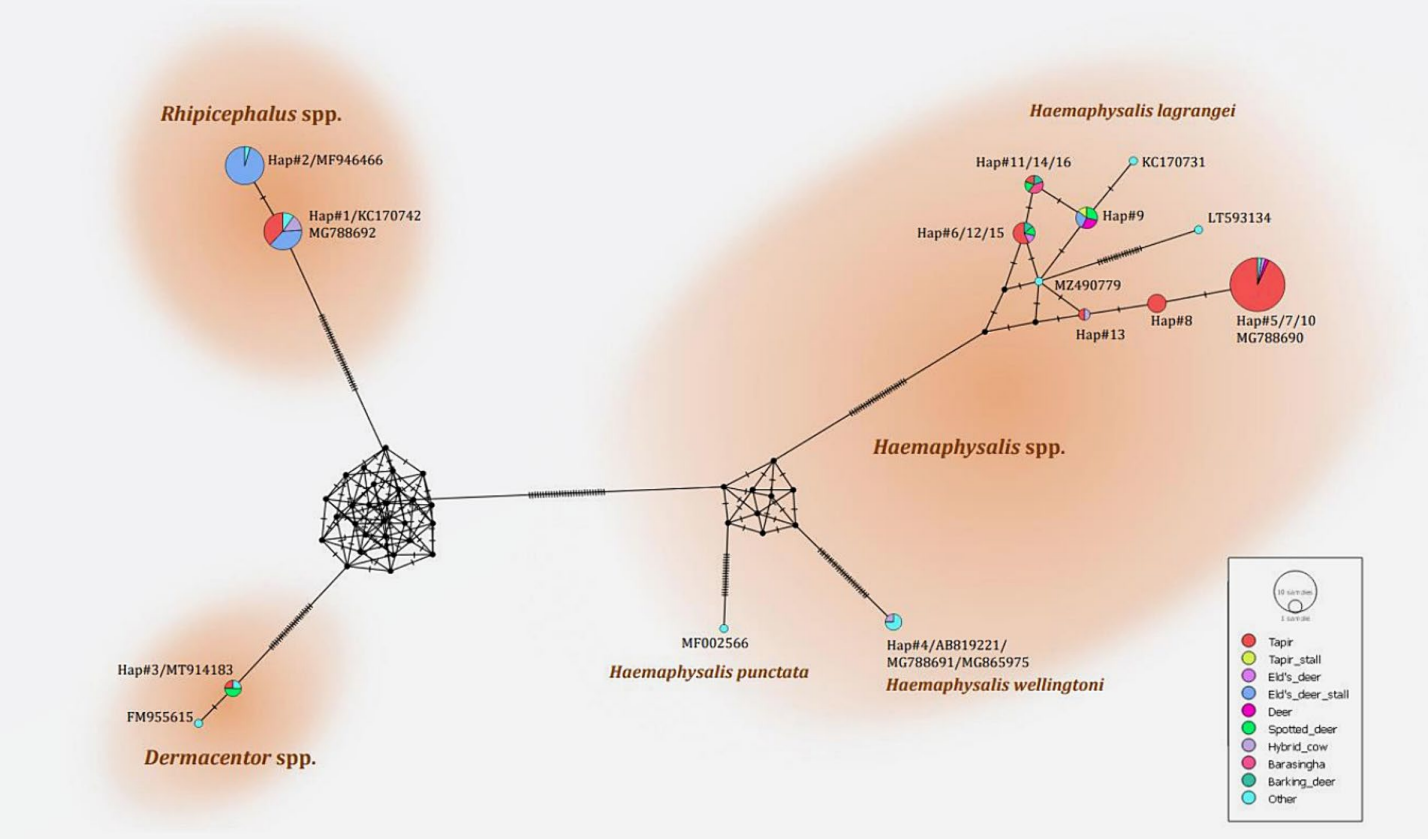
Haplotype networks of the partial mitochondrial *16S rRNA* gene of the tick species identified (413 nucleotide sites) were constructed using a median-joining network in PopART 1.7. Each circle represents a single nucleotide sequence. The size of the circle represents the frequency of each nucleotide sequence, whereas the colors represent the sources or hosts of the ticks collected. “Other” refers to the reference genes.

The phylogenetic tree presented in Figure 2 was generated using the Tamura 3-parameter model (25), derived from *16S rRNA* gene sequences. The phylogenetic tree revealed three groups of tick species: *Haemaphysalis*, *Dermacentor*, and *Rhipicephalus* group. Of the 16 haplotypes, *H. lagrangei* was the most abundant (haplotypes #5–16), followed by *R. microplus* (haplotypes #1–2), *D. auratus* (haplotype #3), and *H. wellingtoni* (haplotype #4). First, *Haemaphysalis* were classified into two clades in which *H. lagrangei, H. bispinosa, H. longicornis* were closer to one another, whereas *H. wellingtoni* was distinct. Second, *Dermacentor* were classified into two clades and our sequences were closer to *D. auratus.* Finally, *Rhipicephalus* were classified into two clades and our sequences were closer to *R. microplus.* The haplotype networks presented in Figure 3 were generated using a median-joining network in PopART 1.7, incorporating a total of 16 haplotypes derived from 114 taxa of *16S rRNA* gene sequences. The network classified the haplotypes into three distinct groups consisting of *Haemaphysalis*, *Dermacentor*, and *Rhipicephalus* group. First, *H. lagrangei* were found in all wildlife hosts and habitats employed in this study and *H. wellingtoni* were found in a hybrid cow. Second, *D. auratus* were found in spotted deer and tapir. Finally, *R. microplus* were found in all Eld’s deer habitats, tapir, and hybrid cow.

#### 3.1.2 Association between season and number of collected ticks

**Figure 4:**
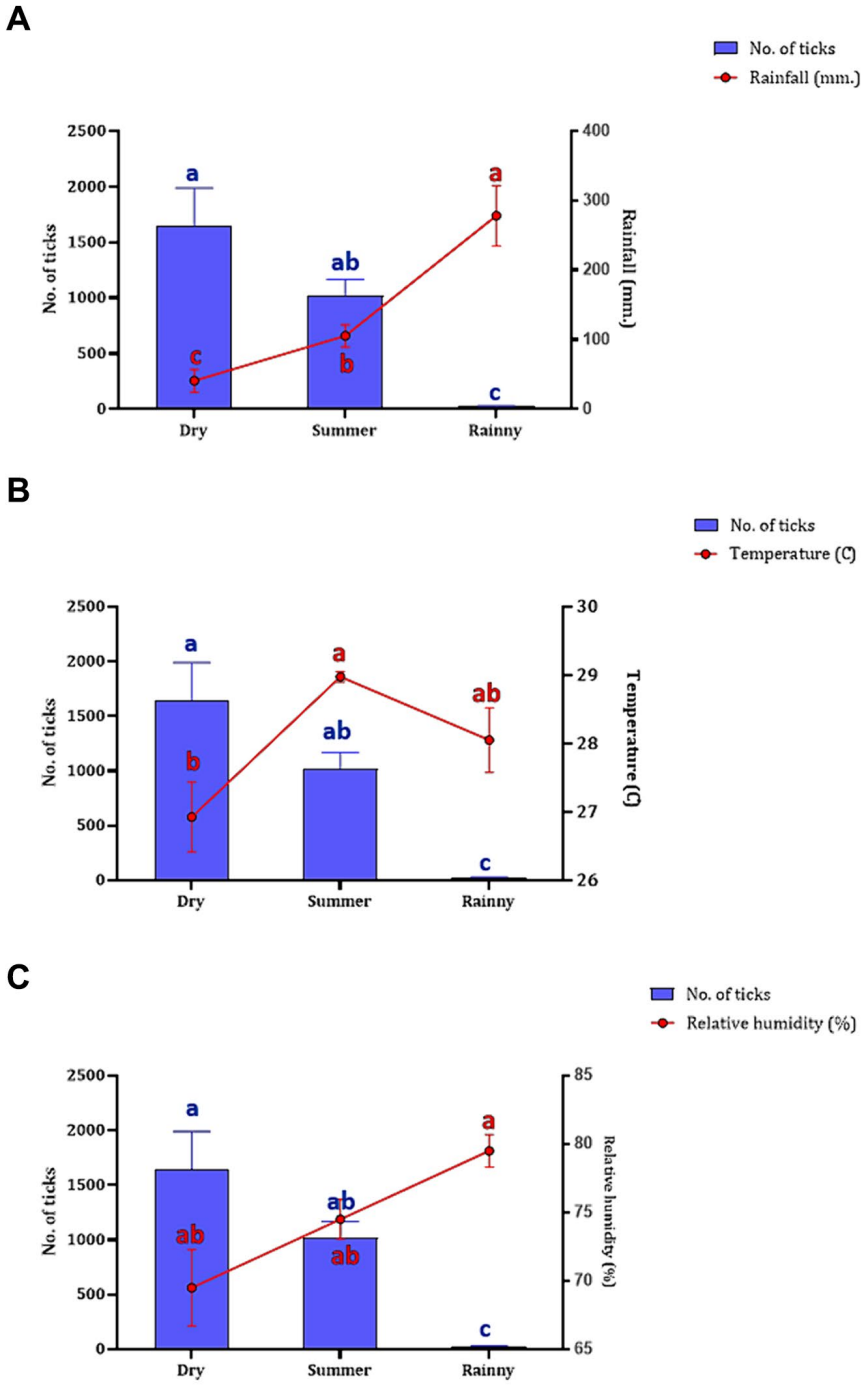
The number of questing ticks collected in each season (represented by blue bar) relative to the weather variables (represented by red circle and line), including rainfall **(A)**, temperature **(B)**, and humidity **(C)**. The seasons in Thailand, namely, summer (collected in March–June 2021), rainy (collected in July–October 2021), and dry (collected in November 2021–February 2022). Data are presented as means with SEM. The blue bars with distinct blue letters and the red circles with distinct red letters denote significant differences between each season in a two-way ANOVA.

The association between the season and number of questing ticks collected by vegetation dragging was determined. Following the seasonal classifications in Thailand,^2^ three seasons were established: summer (March–June), rainy (July–October), and dry season (November–February). Our findings indicated a relationship between rainfall and the collection of questing ticks in the Khao Kheow Open Zoo. Interestingly, the summer and dry seasons exhibited a more pronounced impact on the active questing ticks collected via vegetation dragging in wildlife areas than in the rainy season. To comprehensively assess the influence of weather variables on tick collection, we analyzed the correlation between the number of ticks collected in each season and specific meteorological data such as rainfall, temperature, and relative humidity. We found while the rainfall significantly negatively affected the quantity of ticks collected (Figure 4A), the temperature and the relative humidity did not (Figures 4B,C). This demonstrated that the trend in the number of questing ticks collected varied relative to the amount of rainfall in each season.

### 3.2 Pathogen detection in collected ticks

The overall infection rate for the 449 samples (10,436 ticks) was 66.37% (298/449), with the highest infection rate by Anaplasmataceae family at 55.23% (248/449), followed by *Babesia* at 29.62% (133/449) and *Theileria* at 16.26% (73/449). Of the 449 samples, 114 positive sequences were retrieved which all of which contained pathogens. Among these, 73 sequences, originating from individual tick samples, were analyzed for the prevalence of infection. The highest infection rate in these samples was *Anaplasma* at 100% (73/73), followed by *Theileria* at 9.58% (7/73), and *Babesia* at 5.47% (4/73). The type of infection was classified into single-, co-, or triple-infection. In a single infection, *Anaplasma* was the most abundant at 90.41% (66/73). Nevertheless, no single infection by *Theileria* and *Babesia* was identified. Coinfection of *Anaplasma* and *Theileria* was the most abundant at 5.47% (4/73), followed by *Anaplasma* and *Babesia,* and *Theileria* and *Babesia*, both at 1.36% (1/73). Triple infection of *Anaplasma*, *Theileria*, and *Babesia* was found at 2.73% (2/73). The results are presented in Table 3.

**Table 3:** Prevalence of tick-borne protozoal and bacterial infections in 73 individual tick samples collected from Khao Kheow Open Zoo, Chonburi Province, Thailand.

Tick species	Prevalence of infections (infected/tested samples)			Type of infection						
	*Anaplasma*	*Theileria*	*Babesia*	Single infection			Coinfection			Triple infection
				*Anaplasma*	*Theileria*	*Babesia*	*Anaplasma + Theileria*	*Anaplasma + Babesia*	*Theileria + Babesia*	*Anaplasma + Babesia + Theileria*
*Rhipicephalus microplus*	100% (11/11)	9.09% (1/11)	9.09% (1/11)	90.9% (10/11)	- (0/11)	- (0/11)	- (0/11)	- (0/11)	- (0/11)	9.09% (1/11)
*Dermacentor auratus*	100% (3/3)	66.67% (2/3)	- (0/3)	33.33% (1/3)	- (0/3)	- (0/3)	66.67% (2/3)	- (0/3)	- (0/3)	- (0/3)
*Haemaphysalis wellingtoni*	100% (1/1)	100% (1/1)	- (0/1)	- (0/1)	- (0/1)	- (0/1)	100% (1/1)	- (0/1)	- (0/1)	- (0/1)
*Haemaphysalis lagrangei*	100% (58/58)	5.17% (3/58)	5.17% (3/58)	94.82% (55/58)	- (0/58)	- (0/58)	1.72% (1/58)	1.72% (1/58)	1.72% (1/58)	1.72% (1/58)
Total	100% (73/73)	9.58% (7/73)	5.47% (4/73)	90.41% (66/73)	- (0/73)	- (0/73)	5.47% (4/73)	1.36% (1/73)	1.36% (1/73)	2.73% (2/73)

#### 3.2.1 *Anaplasma* detection and phylogenetic analysis

A chi-square test was performed to determine the parameters associated with the rate of *Anaplasma* detection (Table 4). In terms of tick collection method, we observed a higher detection rate in ticks collected through dragging than through direct sampling (*p*-value = 0.0002). The season of collection was significantly associated with the rate of *Anaplasma* detection, particularly during the dry season (*p*-value = 0.0001). The tick development stage was significantly associated with *Anaplasma* detection, particularly larvae (*p*-value <0.0001). Among adult ticks, *Anaplasma* was more frequently detected in males than in females (*p*-value = 0.0011). The PCR technique employing EHR16SD and EHR16SR primers indicated the presence of *Anaplasma* in 248 of 449 samples (equivalent to 6,971 ticks out of 10,436 ticks). Of these positive samples, 90 samples (1,327 ticks) were utilized for further DNA sequencing. Four species of *Anaplasma*, namely, *A. capra* 90% (81/90), *A. bovis* 6.67% (6/90), unidentified *Anaplasma* spp. 22.22% (2/90), and *A. phagocytophilum* 1.11% (1/90). The nucleotide sequences from the 90 samples were grouped into seven nucleotide sequence types (ntSTs): ntST#1 (two sequences; OQ352827) showing a 100% match with unidentified *Anaplasma* spp. (KY766243); ntST#2 (79 sequences; OQ352818) showing a 100% match with *A. capra* (OQ552619); ntST#3 (one sequence; OQ352831) showing a 100% match with *A. capra* (LC432126); ntST#4 (one sequence; OQ352828) showing a 100% match with *A. capra* (MH762073); ntST#5 (one sequence; OQ352830) showing a 99.67% match with *A. bovis* (MK028574); ntST#6 (5 sequences; OQ352829) showing a 100% match with *A. bovis* (MK028574); and ntST#7 (one sequence; OQ352832) showing a 100% match with *A. phagocytophilum* (MK394178). The results are presented in Table 5.

**Table 4:** The parameters associated with the detection of the Anaplasmataceae family based on PCR diagnostics in tick (Chi-square test; Confidence interval 95%).

Parameters	No. tested samples	Rate of detection (infected/tested samples)	*p*-value
Tick collection method (*n* = 449 samples)			
Dragging	234	63.68% (149/234)	0.0002
Picking	215	46.05% (99/215)	
Season (*n* = 234 samples)			
Summer	129	41.86% (54/129)	<0.0001
Rainy	7	28.57% (2/7)	
Dry	98	94.89% (93/98)	
Stage of tick (*n* = 449 samples)			
Larva	216	66.67% (144/216)	<0.0001
Nymph	51	9.8% (5/51)	
Adult (Female and Male)	182	47.25% (86/182)	
Sex of tick (*n* = 182 samples)			
Female	129	39.53% (51/129)	0.0011
Male	53	66.04% (35/53)	

ns, non-significant; *p-value < 0.05, **p-value < 0.01, ***p-value < 0.001, ****p-value < 0.0001.

**Table 5:** Analysis of protozoal and bacterial nucleotide sequences using the nucleotide Basic Local Alignment Search Tool.

ntST	No. of sequences (*N* = 114)	BLASTn			Length (bp)	Positive tick samples			Vertebrate hosts	Submitted sequences accession no.
		Closest sequence	Species	Identity (%)		No. of sequences	Tick species	Stage		
1	2	KY766243	*Anaplasma* sp.	100	305	1	*Haemaphysalis lagrangei*	F	Tapir	OQ352827
						1	*Dermacentor auratus*	F	Tapir	
2	79	OQ552619	*A. capra*	100	305	13	*H. lagrangei*	F	Tapir	OQ352818
						1	*H. lagrangei*	F	Spotted deer	
						22	*H. lagrangei*	M	Tapir	
						1	*H. lagrangei*	M	Deer	
						1	*H. lagrangei*	M	Spotted deer	
						1	*H. lagrangei*	N	Eld’s deer stall	
						2	*H. lagrangei*	N	Spotted deer	
						15	*H. lagrangei*	L	Tapir stall	
						1	*H. lagrangei*	L	Eld’s deer stall	
						1	*H. wellingtoni*	M	Hybrid cow	
						1	*Rhipicephalus microplus*	M	Hybrid cow	
						5	*R. microplus*	N	Tapir	
						1	*R. microplus*	N	Hybrid cow	
						1	*R. microplus*	N	Eld’s deer	
						1	*R. microplus*	N	Eld’s deer stall	
						3	*R. microplus*	L	Eld’s deer stall	
						8	*R. microplus*	L	Eld’s deer stall	
						1	*D. auratus*	N	Spotted deer	
3	1	LC432126	*A. capra*	100	305	1	*R. microplus*	L	Eld’s deer stall	OQ352831
4	1	MH762073	*A. capra*	100	305	1	*H. lagrangei*	F	Tapir	OQ352828
5	1	MK028574	*A. bovis*	99.67	305	1	*H. lagrangei*	F	Tapir	OQ352830
6	5	MK028574	*A. bovis*	100	305	1	*H. lagrangei*	M	Deer	OQ352829
						1	*H. lagrangei*	F	Deer	
						1	*H. lagrangei*	F	Tapir	
						1	*R. microplus*	F	Tapir	
						1	*D. auratus*	N	Spotted deer	
7	1	MK394178	*A. phagocytophilum*	100	305	1	*R. microplus*	N	Eld’s deer	OQ352832
8	4	KP410273	*Theileria* sp.	97.96	508	1	*R. microplus*	L	Eld’s deer stall	OR003900
						1	*H. lagrangei*	F	Tapir	
						2	*H. lagrangei*	M	Tapir	
9	7	KP410272	*Theileria* sp.	96.47	508	7	*R. microplus*	L	Eld’s deer stall	OR003901
10	2	KP410272	*Theileria* sp.	96.08	508	2	*R. microplus*	L	Eld’s deer stall	OR003902
11	3	KP410272	*Theileria* sp.	96.47	508	3	*R. microplus*	L	Eld’s deer stall	OR003903
12	2	MT463610	*T. equi*	99.71	508	2	*R. microplus*	L	Tapir stall	OR003904
13	6	KY766213	*Babesia* sp.	99.16	479	3	*R. microplus*	L	Eld’s deer stall	OR003905
						1	*H. lagrangei*	F	Tapir	
						2	*H. lagrangei*	M	Tapir	

BLASTn, nucleotide Basic Local Alignment Search Tool. ntST, nucleotide sequence type.

**Figure 5:**
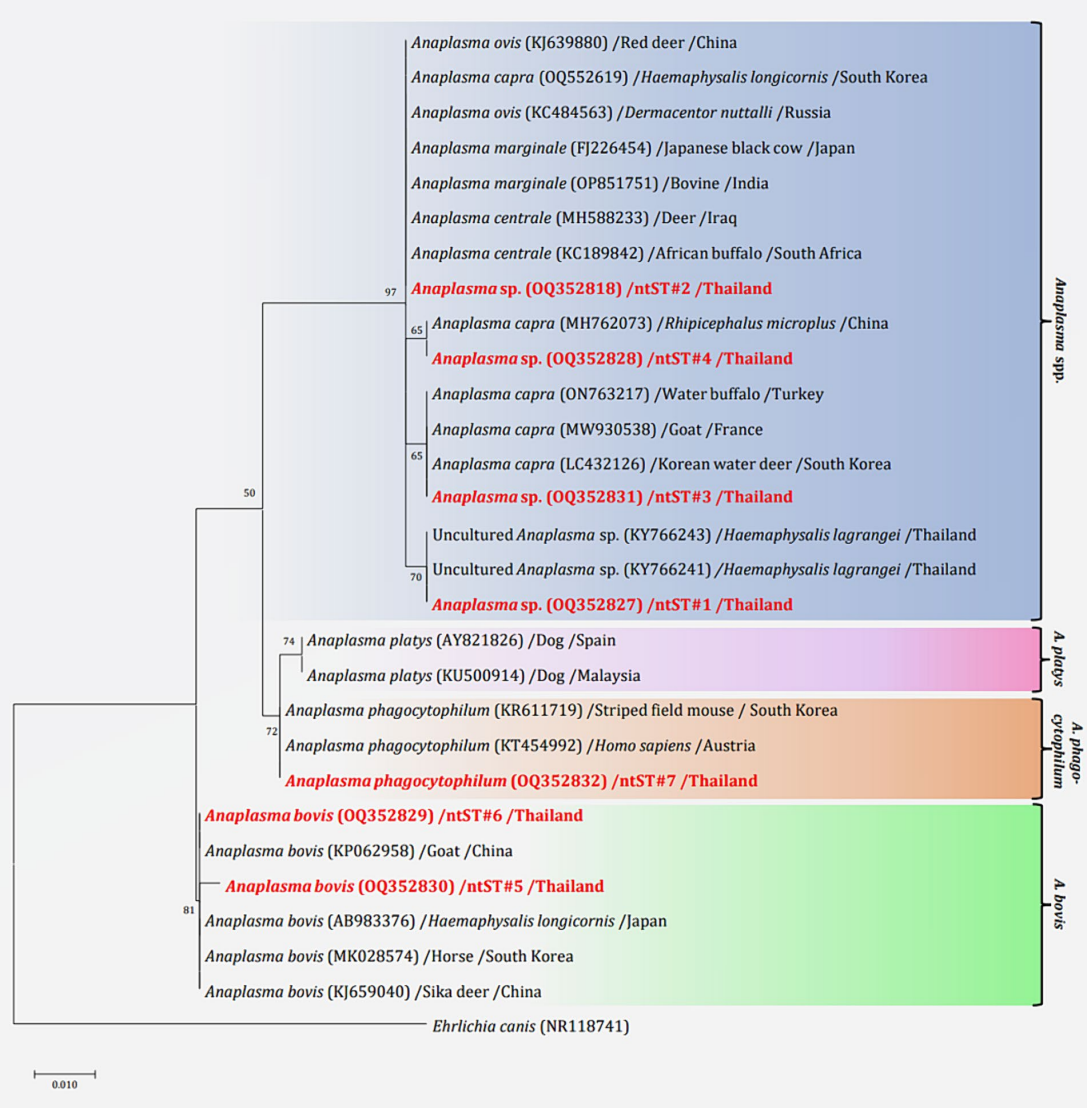
Maximum likelihood phylogenetic tree of 306 nucleotide sites from partial mitochondrial *16S rRNA* genes of the *Anaplasma* spp. found in this study (highlighted in red) and the global isolates. Nucleotide sequences were determined using the Kimura 2-parameter model (K2) with 1,000 bootstrap replications and *Ehrlichia canis* (NR118741) as the outgroup. The tree is drawn to scale, with branch lengths indicating the number of substitutions per site.

**Figure 6:**
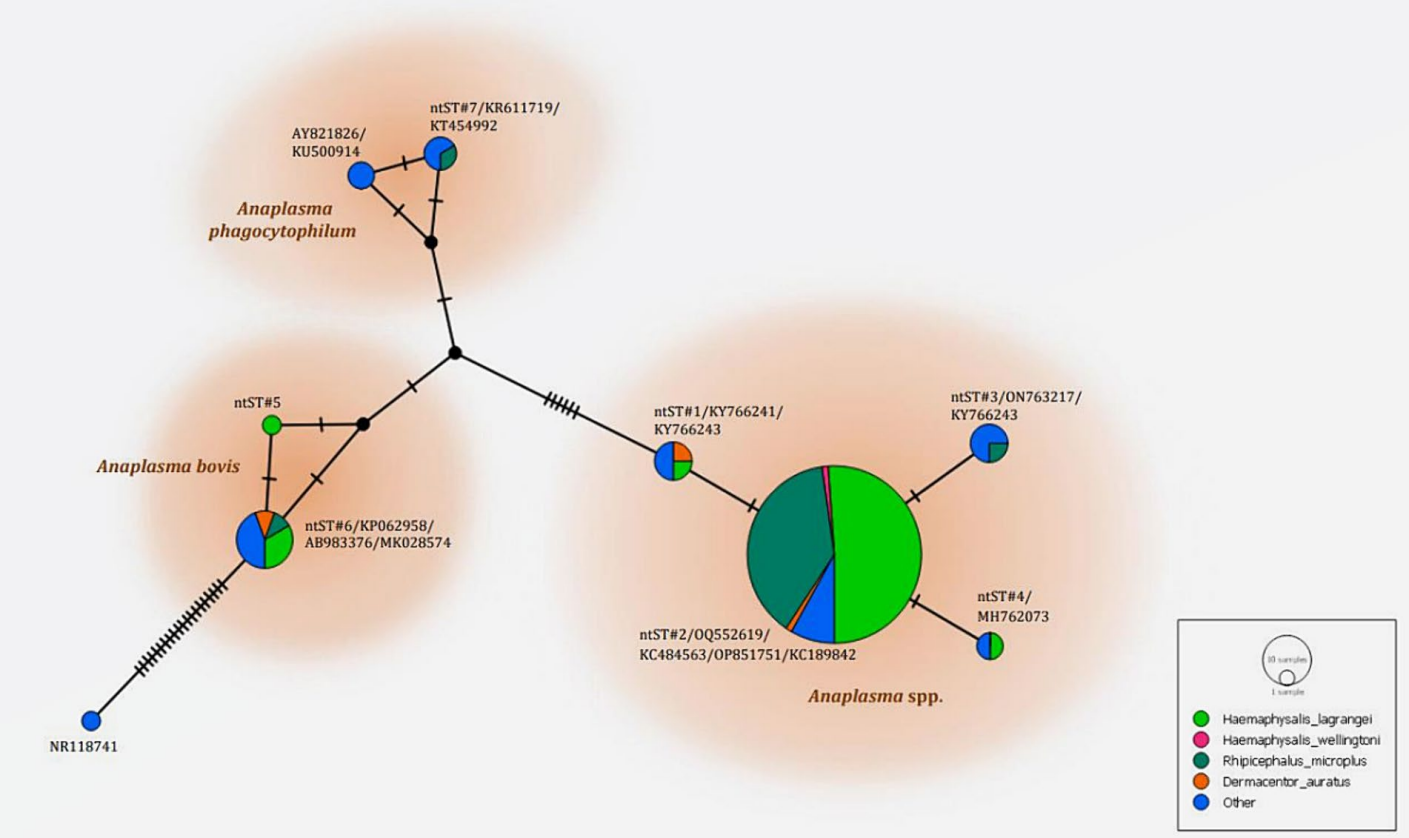
Nucleotide sequence type (ntST) networks of the *16S rRNA* gene (306 nucleotide sites) of *Anaplasma* spp. The network was constructed using a median-joining network in PopART 1.7. Each circle represents a different haplotype. The size of the circle represents the frequency of each ntST, while the colors represent the tick species. “Other” refers to the reference genes.

The phylogenetics presented in Figure 5 were generated using the Kimura 2-parameter model (K2) model (26). The *Anaplasma 16S rRNA* gene sequences were used to generate a phylogenetic to compare them with the sequences for 21 *Anaplasma* strains registered in the GenBank database. The phylogenetics are characterized into three clusters. Eighty-three DNA sequences (ntST#1–4) were placed in the same cluster at 97% bootstrap value. Within this cluster, ntST#2 was categorized among the branches of two *A. centrale* (KC189842 and MH588233), two *A. ovis* (KJ639880 and KC484563), two *A. marginale* (OP851751 and FJ226454), and one *A. capra* (OQ552619). ntST#1 was divergent and was categorized with two *Anaplasma* spp. (KY66243 and KX505303) and ntST#3 (LC432126) and ntST#4 (MH762073) with *A. capra*. One *Anaplasma* DNA sequence of ntST#7 was classified in the same cluster at 72% bootstrap value with two *A. phagocytophilum* (KR611719 and KT454992). Five *Anaplasma* DNA sequences of ntST#6 were placed in the same cluster at 81% bootstrap value with several *A. bovis* (KJ659040, AB983376, KP062958, and MK028574), whereas one sequence ntST#5 was found to be divergent. The networks of nucleotide sequence types presented in Figure 6 were generated using a median-joining network in PopART 1.7 and identified seven haplotypes derived from 90 taxa of *16S rRNA* gene sequences were identified. The network classified the nucleotide sequence types into three distinct groups: *Anaplasma* spp., *A. bovis*, and *A. phagocytophilum*. *Anaplasma* spp. were found in four species of ticks, namely, *H. lagrangei, H. wellingtoni, R. microplus,* and *D. auratus*. Furthermore, *A. bovis* was found in *H. lagrangei, R. microplus,* and *D. auratus*, wheeas *A. phagocytophilum* was only found in *R. microplus*.

#### 3.2.2 *Theileria* and *Babesia* detection and phylogenetic analysis

The chi-square test was performed to determine the parameters associated with the rate of detection of tick species. *Theileria* was observed more frequently in ticks collected by vegetation dragging than by direct sampling (*p*-value = 0.0001; Table 6). Furthermore, we observed a higher incidence of *Theileria* in larvae than that in the other stages (*p*-value <0.0001). However, the rate of *Theileria* detection was not significantly associated with tick sex or season of collection. The rate of *Babesia* detection was significantly associated with the season, particularly the dry season (*p*-value = 0.0069; Table 7). The detection frequency of *Babesia* was significantly higher with vegetation dragging than with direct sampling (*p*-value <0.0001). Additionally, we noted a significantly higher incidence of *Babesia* in larvae compared to other developmental stages (*p*-value <0.0001). Nevertheless, no significant association was observed between tick sex and rate of *Babesia* detection.

**Table 6:** The parameters associated with the detection of the *Theileria* genus based on PCR diagnostics in tick (Chi-square test; Confidence interval 95%).

Parameters	No. tested (samples)	Rate of detection (infected/tested samples)	*p*-value
Tick collection method (*n* = 449 samples)			
Dragging	234	22.65% (53/234)	0.0001
Picking	215	9.3% (20/215)	
Season (*n* = 234 samples)			
Summer	129	20.16% (26/129)	0.5898 (ns)
Rainy	7	28.57% (2/7)	
Dry	98	25.51% (25/98)	
Stage of ticks (*n* = 449 samples)			
Larvae	216	24.07% (52/216)	<0.0001
Nymphs	51	1.96% (1/51)	
Adults (Female and Male)	182	8.79% (16/182)	
Sex of ticks (*n* = 182 samples)			
Females	129	8.53% (11/129)	0.8444 (ns)
Males	53	9.43% (5/53)	

ns, non-significant; *p-value < 0.05, **p-value < 0.01, ***p-value < 0.001, ****p-value < 0.0001.

**Table 7:** The parameters associated with the detection of the *Babesia* genus based on PCR diagnostics in tick (Chi-square test; Confidence interval 95%).

Parameters	No. tested (samples)	Rate of detection (infected/tested samples)	*p*-value
Tick collection method (*n* = 449 samples)			
Dragging	234	48.72% (114/234)	<0.0001
Picking	215	8.84% (19/215)	
Season (*n* = 234 samples)			
Summer	129	48.06% (62/129)	0.0069
Rainy	7	- (0/7)	
Dry	98	53.06% (52/98)	
Stage of ticks (*n* = 449 samples)			
Larvae	216	51.39% (111/216)	<0.0001
Nymphs	51	5.88% (3/51)	
Adults (Female and Male)	182	7.14% (13/182)	
Sex of ticks (*n* = 182 samples)			
Females	129	5.43% (7/129)	0.1607 (ns)
Males	53	11.32% (6/53)	

ns, non-significant; *p-value < 0.05, **p-value < 0.01, ***p-value < 0.001, ****p-value < 0.0001.

The PCR results for *Theileria* spp. (Table 5) using the partial *18S rRNA* gene, 989-F and 990-R primers, revealed that 16.26% (73/449) of the samples were positive for the protozoal species. Two species of *Theileria* were identified including, unidentified *Theileria* spp. 88.89% (16/18) and *T. equi* at 11.11% (2/18). In terms of *Babesia* spp. (Table 5), the PCR results using the partial *18S rRNA* gene, Bab-F and Bab-R primers, revealed that 29.62% (133/449) of the samples were positive for the species. The protozoal DNA sequences were grouped into six ntSTs: ntST#8–11 (16 sequences; OR003900-03) showing a 96.08–97.96% match with *Theileria* spp. (KP410272 and KP410273); ntST#12 (two sequences; OR003904) showing a 99.71% match with *T. equi* (MT463610); and ntST#13 (six sequences; OR003905) showing a 99.16% match with *Babesia* spp. (KY766213). The DNA sequences are available from GenBank.

**Figure 7:**
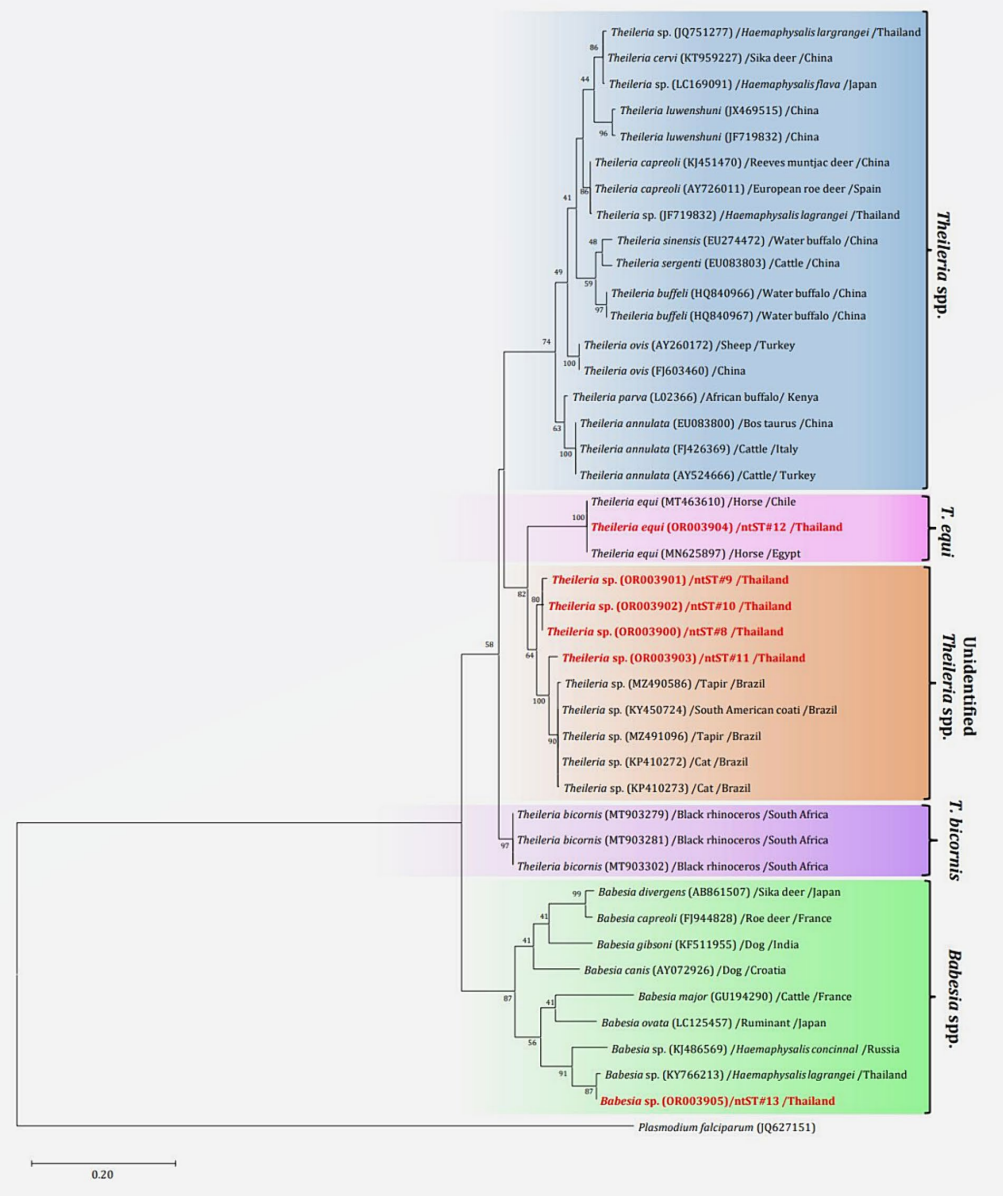
Maximum likelihood phylogenetic tree of the partial *18S rRNA gene* (603 nucleotide sites) of *Theileria* and *Babesia* spp. found in this study (highlighted in red) and the global isolates. Nucleotide sequences were generated using the Tamura–Nei model with 1,000 bootstrap replications, with *Plasmodium falciparum* (JQ627151) as the outgroup. The tree is drawn to scale, with branch lengths indicating the number of substitutions per site.

**Figure 8:**
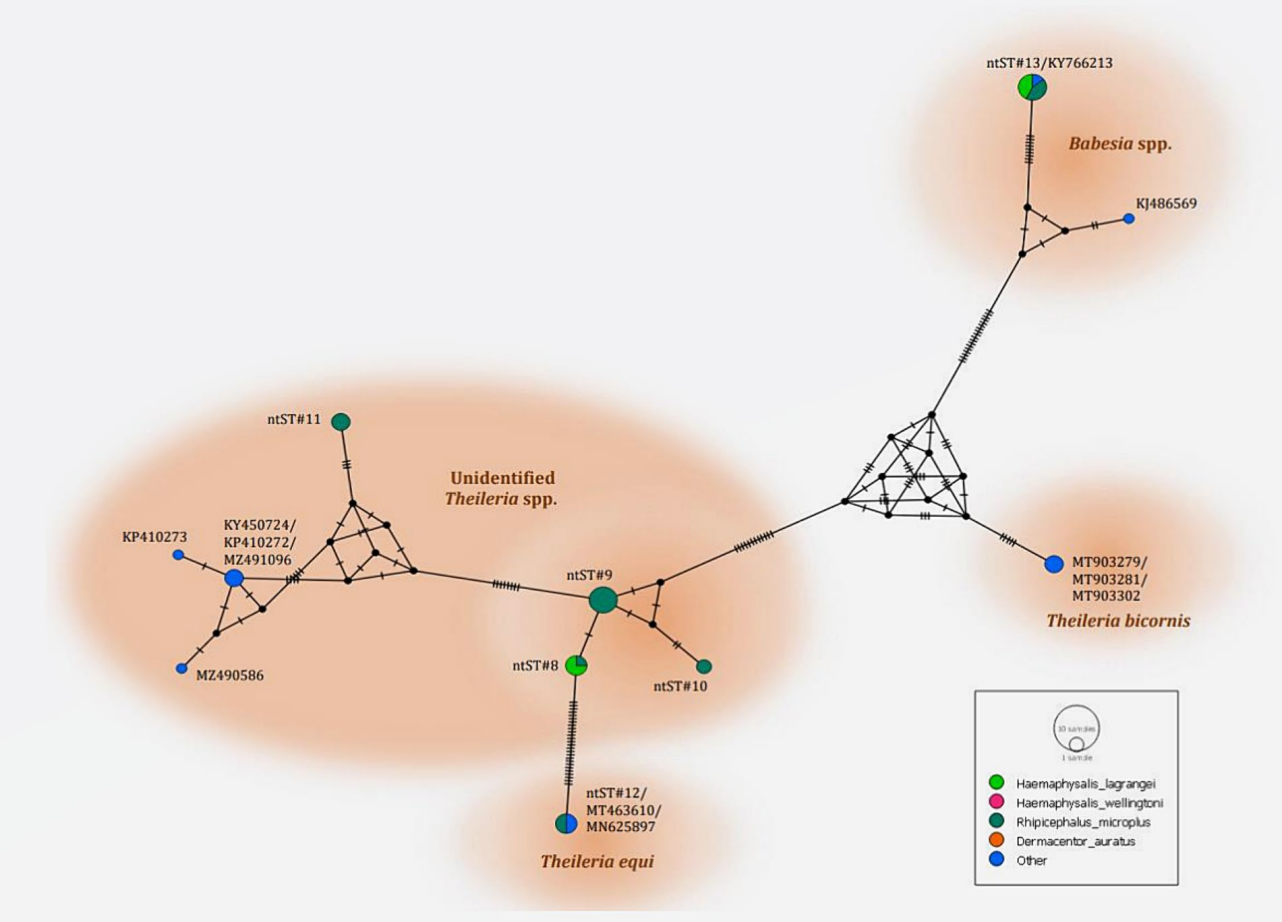
Nucleotide sequence type (ntST) networks of the *18S rRNA* gene (603 nucleotide sites) of *Theileria* and *Babesia* spp. were constructed using the median-joining network function in PopART 1.7. Each circle represents a different haplotype. The size of the circle represents the frequency of each ntST, and the colors represent the tick species identified. “Other” indicates the reference genes.

The phylogenetics presented in Figure 7 were generated using the Tamura-Nei model (27). Analysis of the *18S rRNA* gene sequences of *Theileria* and *Babesia* was performed for comparison with the 36 *Theileria* and *Babesia* strains registered in the GenBank database. The DNA sequences were phylogenetically characterized into four clusters: a putative novel species of 13 DNA sequences (ntST#8–10) classified into the *Theileria* group with the same cluster at 80% bootstrap value; three DNA sequences of ntST#11 categorized in the same cluster at 100% bootstrap value with five *Theileria* spp., although more divergence was found in this sequence; two DNA sequences of ntST#12 categorized in the same cluster at 100% bootstrap value with two *T. equi* (MT463610 and MN625897); and six DNA sequences of ntST#13 classified in the same cluster at 91% bootstrap value with two *Babesia* spp. (KY766213 and KJ486569). The networks of nucleotide sequence types presented in Figure 8 were constructed using the median-joining network function in PopART 1.7 from a total of six ntSTs (24 taxa of *18S rRNA* gene sequences). The nucleotide sequence types were classified into four groups: unidentified *Theileria* spp. were found in two species of ticks, namely, *H. lagrangei,* and *R. microplus*; *Theileria* spp. found in *H. lagrangei*; *T. equi* found in *R. microplus*; and *Babesia* spp. found in *H. lagrangei* and *R. microplus*.

## 4 Discussion

In this study, various protozoa and bacteria species were identified from 10,436 tick specimens collected. The larva was the predominant specimen type, followed by the nymph, female, and male specimens, respectively. Our field data indicated that a higher quantity of ticks can be collected during the dry season (November–February) at the Khao Keow Open Zoo in Thailand. Conversely, in the Amazon, tick density is higher during the rainy season (July–November) (28). We also observed significant detection rates for tick pathogens during the dry season. This concurs with a previous study in 2023 that also observed significant infection rates of *Anaplasma* spp. in beef cattle during the dry season in Thailand, which was linked to ticks or blood-sucking flies (29). Since animals and wildlife in the open zoo have free access to the wild, the high tick infection rates in our study could be partially explained by interactions with wildlife.

Four tick species, namely, *H. lagrangei*, *H. wellingtoni*, *D. auratus*, and *R. microplus*, were identified in this present study, which confirmed the previous reports of tick species occurring in many parts of Thailand (15, 30, 31). Ticks play a crucial role as ectoparasites affecting wildlife, livestock, and companion animals in Thailand (32, 33). The present study found that all four tick species (*H. lagrangei*, *H. wellingtoni*, *D. auratus*, and *R. microplus*) were all infected by bacteria in the genus *Anaplasma*, including unidentified *Anaplasma* spp., *A. phagocytophilum,* and *A. bovis.* Based on the results of *16S rRNA* gene sequencing, the harbored *Anaplasma* species in this study matched with *A. marginale*, *A. ovis*, *A. centrale*, and *A. capra*, which were collected from tapirs, deer, spotted deer, Eld’s deer, hybrid cows, and vegetation. A previous study based on the results of *16S rRNA* gene sequencing reported four *Anaplasma* spp. (*A. marginale*, *A. bovis*, *A. phagocytophilum*, and *A. centrale*) detected in *R. microplus* specimens collected from tapirs, cows, and surrounding vegetation (34). Nevertheless, limitations were found when using the *16S rRNA* sequence for species classification of *Anaplasma* samples (35). The PCR primers used in the present study, specifically designed for amplification of the *16S rRNA* gene, showed limited accuracy in distinguishing *Anaplasma* isolates at the species level. Careful consideration needs to be exercised in the design of PCR primers by incorporating the alignments of diverse target genes and alternative genetic markers. This strategic approach is imperative for improving the precision and specificity of identifying and characterizing *Anaplasma* species.

We identified *A. phagocytophilum* in the *R. microplus* ticks collected from Eld’s deer. This finding aligns with the observations of Zhang et al. (36), who reported *A. phagocytophilum* infection in *R. microplus* ticks collected from 10 provinces in China. This important human pathogen has a broad host range and can cause severe infections in various mammalian species (37). Furthermore, we identified *Anaplasma bovis* in *H. lagrangei, R. microplus*, and *D. auratus* ticks collected from deer, spotted deer, and tapir. *A. bovis* causes diseases in both ruminants and small mammals, with transmission facilitated by *Haemaphysalis, Rhipicephalus, Amblyomma*, and *Ixodes* (7). Previous studies have identified certain species of *Haemaphysalis,* including *H. lagrangei, H. megaspinosa*, and *H. longicornis*, as potential vectors of *A. bovis* in East and Southeast Asia (31, 38). Given the widespread distribution of these tick species in Southeast Asia, infection of domestic cattle and wildlife, including various deer species, poses a significant concern (2). While most studies have confirmed *A. bovis* infection across a diverse range of ruminant hosts, its presence in ticks collected from tapirs has not been previously reported. Confirming *A. bovis* infection in ticks obtained from tapirs is crucial to determine whether the ticks acquired the pathogen from the tapir or if they were already harboring the pathogen before contact.

The present study found that *H. lagrangei* and *R. microplus* were infected with *Theileria* and *Babesia 18S rRNA* gene sequencing revealed *Theileria* and *Babesia* spp. in ticks collected from tapir and vegetation. Another study that performed *18S rRNA* gene sequencing reported *Theileria* and *Babesia* spp. in *H. lagrangei* (15). Furthermore, we discovered that some sequences of unidentified *Theileria* spp. were found in the sister clade of *T. equi*. However, the bootstrap value was relatively strong; hence, the sequences might indicate putative novel species, and other gene markers should be considered. In Thailand, *T. equi* is a tick-borne parasite that is considered endemic in equines and mules (39). However, the present study confirmed the natural occurrence of *T. equi* in *R. microplus* collected from tapirs in Chonburi, Thailand. To the best of our knowledge, the occurrence of *T. equi* and closely related genotypes in Thailand has not been previously reported in tapirs or ticks removed from tapirs. Nevertheless, this protozoan species naturally occurs in the South American tapir in Brazil (40). The housing of tapirs in Khao Kheow open zoo areas connects to wildlife sanctuary areas in Thailand, which may promote close contact between different animal species, vector sharing, and consequently pathogen transmission. Thus, our findings corroborate the cross-transmission of pathogens between domestic and wild animals and provide evidence of ticks as possible vectors for diseases in Thailand’s wildlife habitats. Although we detected *Theileria* in tick larvae, this study could not confirm the transovarial transmission of *Theileria*. Additional evidence is required to address this question. However, our findings align with those of Wattanamethanont et al. (15), who identified *Theileria* in the larvae of ixodid ticks collected by vegetation dragging in a national park in Thailand. These findings imply that ixodid ticks actively searching for hosts in wildlife habitats might be potential vectors for *Theileria* in Thailand.

Interestingly, our findings demonstrated coinfection with *Anaplasma* spp., *Theileria* spp., and *Babesia* spp. Ticks infesting wild animals in their natural habitats can become co-infected, transmitting two or more pathogens (41). Co-infection with more than one tick-borne pathogen is a common occurrence that amplifies pathogenic processes and consequently increases the risk of disease severity (11, 42). Furthermore, pathogen interactions can also increase the risk of infection in wildlife (43). Despite the lack of blood samples collected from wildlife to assess for infection in the present study, data on the various pathogens in ticks observed in this study can be used to assess the well-being of wildlife and monitor diseases. Additionally, the cross-transmission of ticks between humans and wildlife increased with the rise in outdoor human activities in natural wildlife habitats. Thus, awareness of the risks of zoonotic diseases should be increased. Our findings also demonstrated that certain tick species found in Thailand are possible vectors of tick-borne diseases in wildlife at the Khao Kheow Open Zoo, as confirmed by the detection of pathogens in the areas studied. The present study provided valuable insights for the effective treatment, prevention, and planning of annual tick control and surveillance in open zoo areas to prevent tick-borne illnesses. However, additional research is required to determine the ability of each species to transmit such diseases and to enhance the understanding of the relationships among pathogens, ticks, and hosts.

## Data availability statement

The data supporting the results of this study can be obtained from the corresponding author upon request. Moreover, all DNA sequences have been deposited in GenBank® under the following accession numbers: the 16S rRNA gene from collected ticks (OQ918450-OQ918465), the 16S rRNA gene obtained from Anaplasma isolates (OQ352818, OQ352827-OQ352832), and the 18S rRNA gene obtained from protozoa isolates (OR003900-OR003905).

## Ethics statement

The animal study was approved by Chulalongkorn University Animal Care and Use Committee (Animal Use Protocol no. 2131007 and 2231058) and the Animal Conservation and Research Institute Committee, Zoological Park Organization of Thailand. The study was conducted in accordance with the local legislation and institutional requirements.

## Author contributions

CS: Investigation, Methodology, Writing – original draft. KT: Investigation, Writing – review & editing. WW: Investigation, Writing – review & editing. NY: Investigation, Writing – review & editing. GR: Investigation, Writing – review & editing. CA: Investigation, Writing – review & editing. NB: Investigation, Writing – review & editing. NS: Investigation, Writing – review & editing. ER: Investigation, Writing – review & editing. TB: Investigation, Writing – review & editing. PaK: Investigation, Writing – review & editing. UM: Investigation, Writing – review & editing. PiK: Investigation, Writing – review & editing. AS: Investigation, Writing – review & editing. LB: Writing – review & editing. ST: Funding acquisition, Methodology, Supervision, Writing – review & editing.

## Footnotes

^1^ http://www.ub.edu/dnasp/

^2^ www.tmd.go.th

## Supplementary material

SUPPLEMENTARY FIGURE 1 Parts of whole tick collection methods **(A)**, tick stages **(B)**, and pool samples 622 **(C)** established in this study.
