# Supplementary figures and images for: Tick diversity and molecular detection of Anaplasma, Babesia, and Theileria from Khao Kheow open zoo, Chonburi Province, Thailand
Source: Front Vet Sci. 2024 Jul 1;11:1430892. doi: 10.3389/fvets.2024.1430892 (PMC11250040; doi:10.3389/fvets.2024.1430892)

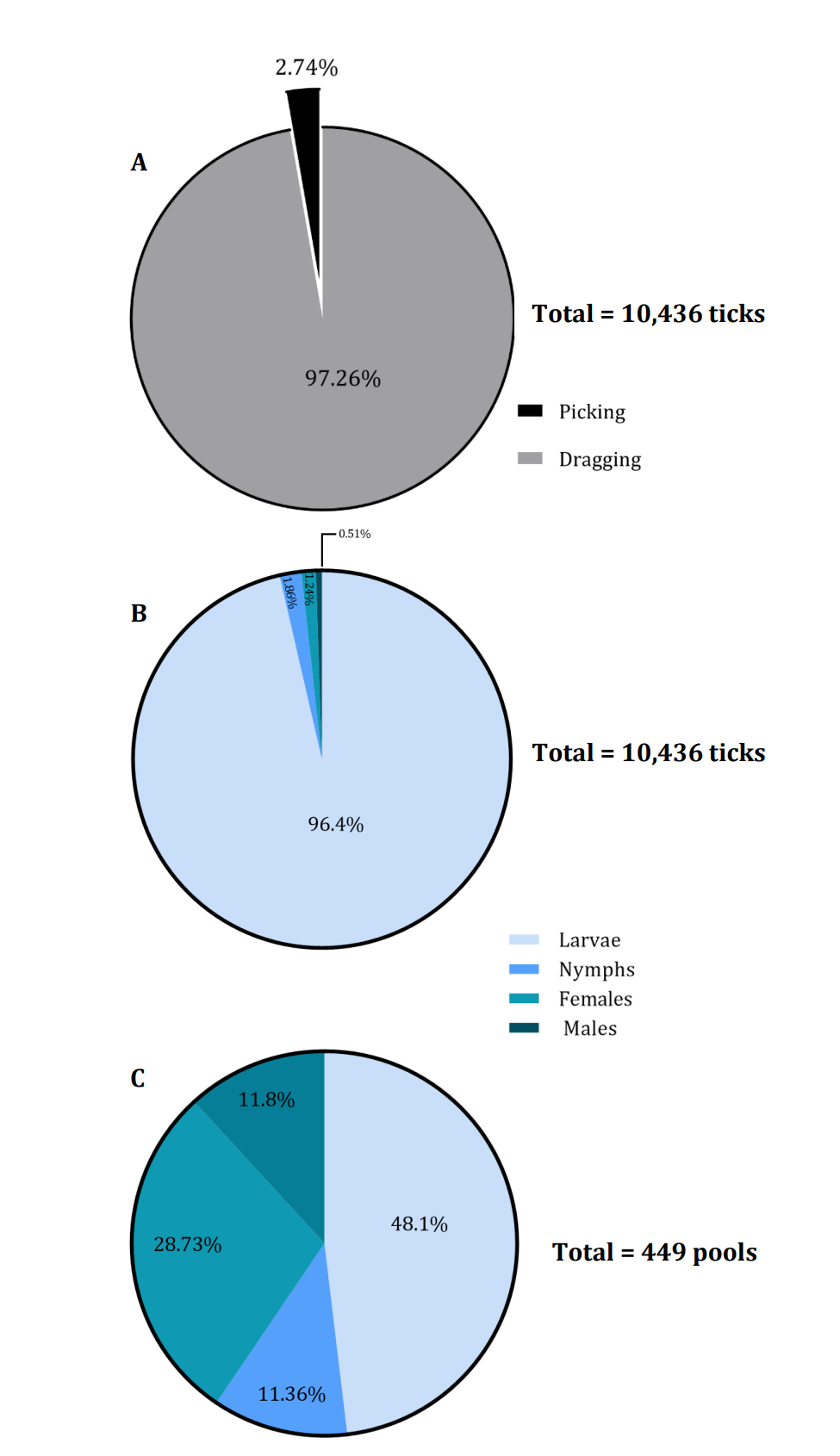

Supplement: SUPPLEMENTARY FIGURE 1 — Parts of whole tick collection methods (A), tick stages (B), and pool samples 622 (C) established in this study. [file Image_1.TIFF]
