# Supplementary material for: Tick diversity and molecular detection of Anaplasma, Babesia, and Theileria from Khao Kheow open zoo, Chonburi Province, Thailand
Source: Front Vet Sci. 2024 Jul 1;11:1430892. doi: 10.3389/fvets.2024.1430892 (PMC11250040; doi:10.3389/fvets.2024.1430892)
Supplement: Supplementary file 2 [file Data_Sheet_1.PDF]

Supplementary Table 1

Label (pool samples)	Tick's stage	Methods	Host/Habitat	No. of ticks	Primer: EHR 16SD/16SR (Anaplasmataceae)	Primer: 989-F/990-R (*Theileria* spp.)	Primer: Bab-F/Bab-R (*Babesia* spp.)
001	Larvae	Dragging	Tapir (stall)	50	0	0	0
002	Larvae	Dragging	Tapir (stall)	50	0	0	0
003	Larvae	Dragging	Tapir (stall)	50	0	0	0
004	Larvae	Dragging	Tapir (stall)	50	0	0	0
005	Larvae	Dragging	Tapir (stall)	50	0	0	0
006	Larvae	Dragging	Tapir (stall)	50	0	0	0
007	Larvae	Dragging	Tapir (stall)	50	0	0	0
008	Larvae	Dragging	Tapir (stall)	50	0	0	0
009	Larvae	Dragging	Tapir (stall)	50	0	0	0
010	Larvae	Dragging	Tapir (stall)	50	0	0	0
011	Larvae	Dragging	Tapir (stall)	50	0	0	0
012	Larvae	Dragging	Tapir (stall)	5	0	0	0
013	Larvae	Dragging	Tapir (stall)	1	1	0	0
014	Larvae	Dragging	Male eld's deer (stall)	50	0	0	0
015	Larvae	Dragging	Male eld's deer (stall)	50	0	0	0
016	Larvae	Dragging	Male eld's deer (stall)	50	0	0	0
017	Larvae	Dragging	Male eld's deer (stall)	40	0	0	0
018	Larvae	Dragging	Male eld's deer (stall)	3	0	0	0
019	Nymph	Dragging	Tapir (stall)	10	0	0	0
020	Nymph	Dragging	Tapir (stall)	1	0	0	0
021	Nymph	Dragging	Male eld's deer (stall)	1	0	0	0
022	Nymph	Picking	Tapir	1	0	0	0
023	Nymph	Picking	Tapir	1	0	0	0
024	Nymph	Picking	Tapir	1	0	0	0

025	Nymph	Picking	Tapir	1	0	0	0
026	Nymph	Picking	Tapir	1	1	0	0
027	Nymph	Picking	Tapir	1	1	0	1
028	Nymph	Picking	Tapir	1	1	0	0
029	Nymph	Picking	Tapir	1	0	0	0
030	Nymph	Picking	Tapir	1	0	0	0
031	Nymph	Picking	Tapir	1	0	0	0
032	Nymph	Picking	Tapir	1	1	0	0
033	Nymph	Picking	Tapir	1	1	0	0
034	M	Picking	Tapir	1	1	0	0
035	F	Picking	Tapir	1	1	0	0
036	F	Picking	Tapir	1	1	0	0
037	Larvae	Dragging	Tapir (stall)	48	0	0	0
038	Larvae	Dragging	Male eld's deer (stall)	50	0	0	1
039	Larvae	Dragging	Male eld's deer (stall)	50	0	1	0
040	Larvae	Dragging	Male eld's deer (stall)	50	0	0	0
041	Larvae	Dragging	Male eld's deer (stall)	50	0	1	1
042	Larvae	Dragging	Male eld's deer (stall)	50	0	0	1
043	Larvae	Dragging	Male eld's deer (stall)	50	0	0	1
044	Larvae	Dragging	Male eld's deer (stall)	50	0	0	1
045	Larvae	Dragging	Male eld's deer (stall)	50	0	0	1
046	Larvae	Dragging	Male eld's deer (stall)	50	0	0	1
047	Larvae	Dragging	Male eld's deer (stall)	50	0	0	1
048	Larvae	Dragging	Male eld's deer (stall)	21	0	0	0
049	Larvae	Dragging	Male eld's deer (stall)	10	0	0	0
050	Larvae	Dragging	Male eld's deer (stall)	50	0	0	0
051	Larvae	Dragging	Male eld's deer (stall)	50	0	0	1
052	Larvae	Dragging	Male eld's deer (stall)	50	0	0	1
053	Larvae	Dragging	Male eld's deer (stall)	50	0	0	0

054	Nymph	Dragging	Tapir (stall)	2	0	0	1
055	Nymph	Dragging	Male eld's deer (stall)	1	1	1	1
056	M	Picking	Tapir	1	1	0	0
057	M	Picking	Tapir	1	1	0	0
058	F	Picking	Tapir	1	1	0	0
059	F	Picking	Tapir	1	1	0	0
060	M	Picking	Tapir	1	1	0	0
061	F	Picking	Tapir	1	1	0	0
062	F	Picking	Tapir	1	1	0	0
063	F	Picking	Tapir	1	1	0	0
064	M	Picking	Tapir	1	1	0	0
065	F	Picking	Tapir	1	1	0	0
066	F	Picking	Tapir	1	1	0	0
067	F	Picking	Tapir	1	1	0	0
068	M	Picking	Tapir	1	1	0	0
069	M	Picking	Tapir	1	1	0	0
070	F	Picking	Tapir	1	1	0	0
071	F	Picking	Tapir	1	1	0	0
072	F	Picking	Tapir	1	1	0	0
073	F	Picking	Tapir	1	1	0	0
074	F	Picking	Tapir	1	1	0	0
075	F	Picking	Tapir	1	1	0	0
076	F	Picking	Tapir	1	1	0	0
077	F	Picking	Tapir	1	1	0	0
078	M	Picking	Tapir	1	1	0	0
079	F	Picking	Tapir	1	1	0	0
080	F	Picking	Tapir	1	1	0	0
081	F	Picking	Tapir	1	1	0	0
082	F	Picking	Tapir	1	1	0	0

083	F	Picking	Tapir	1	1	0	0
084	F	Picking	Tapir	1	1	0	0
085	F	Picking	Tapir	1	1	0	0
086	F	Picking	Tapir	1	1	0	0
087	F	Picking	Tapir	1	1	0	0
088	F	Picking	Tapir	1	1	0	0
089	F	Picking	Tapir	1	1	0	0
090	F	Picking	Tapir	1	1	0	0
091	F	Picking	Tapir	1	1	0	0
092	F	Picking	Tapir	1	1	0	0
093	F	Picking	Deer	1	1	0	0
094	F	Picking	Deer	1	1	0	0
095	M	Picking	Deer	1	0	0	0
096	M	Picking	Deer	1	1	0	0
097	M	Picking	Deer	1	1	0	0
098	M	Picking	Deer	1	0	0	0
099	M	Picking	Deer	1	1	0	0
100	F	Picking	Deer	1	0	0	0
101	F	Picking	Deer	1	1	0	0
102	M	Picking	Female Barasingha	1	0	0	0
103	F	Picking	Female Barasingha	1	0	0	0
104	F	Picking	Female Barasingha	1	0	0	0
105	F	Picking	Female Barasingha	1	0	0	0
106	F	Picking	Female Barasingha	1	0	0	0
107	F	Picking	Female Barasingha	1	0	0	0
108	F	Picking	Female Barasingha	1	0	0	0
109	Nymph	Picking	Male Barasingha	3	0	0	0
110	Nymph	Picking	Male Barasingha	1	0	0	0
111	F	Picking	Male Barasingha	1	0	0	0

112	F	Picking	Male Barasingha	1	0	0	0
113	M	Picking	Male Barasingha	1	1	0	0
114	F	Picking	Male Barasingha	1	0	0	0
115	M	Picking	Male Barasingha	1	0	0	0
116	F	Picking	Male Barasingha	1	0	0	0
117	F	Picking	Male Barasingha	1	0	0	0
118	F	Picking	Male Barasingha	1	1	0	0
119	M	Picking	Male Barasingha	1	0	0	0
120	M	Picking	Male Barasingha	1	0	0	0
121	F	Picking	Male Barasingha	1	0	0	0
122	Larvae	Dragging	Male eld's deer (stall)	50	0	0	0
123	Larvae	Dragging	Male eld's deer (stall)	50	1	0	0
124	Larvae	Dragging	Male eld's deer (stall)	50	1	1	1
125	Larvae	Dragging	Male eld's deer (stall)	50	0	0	1
126	Larvae	Dragging	Male eld's deer (stall)	50	0	1	1
127	Larvae	Dragging	Male eld's deer (stall)	50	1	1	1
128	Larvae	Dragging	Male eld's deer (stall)	50	0	1	1
129	Larvae	Dragging	Male eld's deer (stall)	50	0	1	1
130	Larvae	Dragging	Male eld's deer (stall)	50	0	1	1
131	Larvae	Dragging	Male eld's deer (stall)	19	1	0	1
132	Larvae	Dragging	Tapir	6	0	0	1
133	Nymph	Picking	Male eld's deer (stall)	1	1	0	0
134	Nymph	Picking	Tapir	1	1	0	0
135	Nymph	Picking	Male eld's deer (stall)	9	1	0	0
136	Nymph	Picking	Male eld's deer (stall)	10	0	0	1
137	Nymph	Picking	Male eld's deer (stall)	10	0	0	1
138	Nymph	Picking	Male eld's deer (stall)	11	0	0	1
139	F	Picking	Male eld's deer (stall)	1	0	0	0
140	F	Picking	Male eld's deer (stall)	1	0	0	0

141	F	Picking	Male eld's deer (stall)	1	0	0	0
142	Nymph	Picking	Spotted deer	1	1	1	0
143	Nymph	Picking	Spotted deer	1	1	1	0
144	Nymph	Picking	Spotted deer	1	1	0	0
145	Nymph	Picking	Spotted deer	1	1	1	0
146	F	Picking	Spotted deer	1	0	0	0
147	F	Picking	Spotted deer	1	1	0	0
148	F	Picking	Spotted deer	1	1	0	0
149	F	Picking	Spotted deer	1	0	0	0
150	F	Picking	Spotted deer	1	0	0	0
151	F	Picking	Deer	1	0	0	0
152	F	Picking	Deer	1	1	0	0
153	M	Picking	Deer	1	0	0	0
154	M	Picking	Deer	1	1	0	0
155	F	Picking	Deer	1	0	0	0
156	Larvae	Dragging	Male eld's deer (stall)	50	0	0	1
157	Larvae	Dragging	Male eld's deer (stall)	50	0	0	0
158	Larvae	Dragging	Male eld's deer (stall)	50	1	0	1
159	Larvae	Dragging	Male eld's deer (stall)	50	1	0	1
160	Larvae	Dragging	Male eld's deer (stall)	50	1	0	1
161	Larvae	Dragging	Male eld's deer (stall)	50	1	1	1
162	Larvae	Dragging	Male eld's deer (stall)	50	1	1	1
163	Larvae	Dragging	Male eld's deer (stall)	50	1	0	1
164	Larvae	Dragging	Male eld's deer (stall)	50	1	1	1
165	Larvae	Dragging	Male eld's deer (stall)	50	1	0	1
166	Larvae	Dragging	Male eld's deer (stall)	50	1	0	1
167	Larvae	Dragging	Male eld's deer (stall)	50	1	0	1
168	Larvae	Dragging	Male eld's deer (stall)	50	1	0	1
169	Larvae	Dragging	Male eld's deer (stall)	50	1	1	0

170	Larvae	Dragging	Male eld's deer (stall)	50	1	0	1
171	Larvae	Dragging	Male eld's deer (stall)	50	0	0	1
172	Larvae	Dragging	Male eld's deer (stall)	50	0	0	1
173	Larvae	Dragging	Male eld's deer (stall)	50	0	0	1
174	Larvae	Dragging	Male eld's deer (stall)	50	0	0	1
175	Larvae	Dragging	Male eld's deer (stall)	50	0	0	1
176	Larvae	Dragging	Male eld's deer (stall)	50	1	1	1
177	Larvae	Dragging	Tapir	4	1	0	1
178	Larvae	Dragging	Male eld's deer (stall)	50	1	0	1
179	Larvae	Dragging	Male eld's deer (stall)	50	1	0	1
180	Larvae	Dragging	Male eld's deer (stall)	50	1	0	1
181	Larvae	Dragging	Male eld's deer (stall)	50	1	0	1
182	Larvae	Dragging	Male eld's deer (stall)	50	1	0	1
183	Larvae	Dragging	Male eld's deer (stall)	50	1	1	1
184	Larvae	Dragging	Male eld's deer (stall)	50	1	1	1
185	Larvae	Dragging	Male eld's deer (stall)	50	1	0	1
186	Larvae	Dragging	Male eld's deer (stall)	50	1	0	1
187	Larvae	Dragging	Male eld's deer (stall)	50	1	0	1
188	Larvae	Dragging	Male eld's deer (stall)	50	1	1	1
189	Larvae	Dragging	Male eld's deer (stall)	50	1	1	1
190	Larvae	Dragging	Male eld's deer (stall)	50	1	1	1
191	Larvae	Dragging	Male eld's deer (stall)	50	1	1	1
192	Larvae	Dragging	Male eld's deer (stall)	50	1	1	1
193	Larvae	Dragging	Male eld's deer (stall)	50	1	1	1
194	Larvae	Dragging	Male eld's deer (stall)	50	1	1	1
195	Larvae	Dragging	Male eld's deer (stall)	50	1	1	1
196	Larvae	Dragging	Male eld's deer (stall)	50	1	1	1
197	Larvae	Dragging	Male eld's deer (stall)	50	1	1	1
198	Larvae	Dragging	Male eld's deer (stall)	5	1	0	1

199	Larvae	Dragging	Male eld's deer (stall)	3	1	1	0
200	Larvae	Dragging	Male eld's deer (stall)	34	1	1	0
201	F	Picking	Rusa deer	1	0	0	0
202	F	Picking	Rusa deer	1	0	0	0
203	F	Picking	Rusa deer	1	0	0	0
204	F	Picking	Rusa deer	1	0	0	0
205	Nymph	Dragging	Tapir	3	0	0	0
206	Nymph	Dragging	Tapir	9	0	0	0
207	Larvae	Dragging	Tapir	14	0	0	0
208	Larvae	Dragging	Tapir	44	0	0	0
209	Larvae	Dragging	Tapir	1	0	0	0
210	F	Picking	Deer	1	0	0	0
211	F	Picking	Deer	1	0	0	0
212	F	Picking	Deer	1	0	0	0
213	M	Picking	Deer	1	0	0	0
214	F	Picking	Male eld's deer	1	0	0	0
215	F	Picking	Male eld's deer	1	0	0	0
216	Nymph	Picking	Female barking deer	10	0	0	0
217	Nymph	Picking	Female barking deer	2	0	0	0
218	Nymph	Picking	Male eld's deer	1	0	0	0
219	F	Picking	Male hybrid cow	1	0	0	0
220	F	Picking	Male hybrid cow	1	0	0	0
221	F	Picking	Male hybrid cow	1	0	0	0
222	F	Picking	Male hybrid cow	1	1	0	0
223	M	Picking	Male hybrid cow	1	1	1	0
224	M	Picking	Male hybrid cow	1	1	1	1
225	F	Picking	Tapir	1	1	0	0
226	F	Picking	Tapir	1	1	0	0
227	F	Picking	Tapir	1	1	0	0

228	M	Picking	Tapir	1	1	0	0
229	F	Picking	Tapir	1	1	0	0
230	F	Picking	Tapir	1	0	0	0
231	F	Picking	Tapir	1	0	0	0
232	F	Picking	Tapir	1	0	0	0
233	F	Picking	Tapir	1	0	0	0
234	M	Picking	Tapir	1	1	0	0
235	M	Picking	Tapir	1	1	0	0
236	M	Picking	Tapir	1	0	0	0
237	F	Picking	Tapir	1	0	0	0
238	M	Picking	Tapir	1	0	0	0
239	M	Picking	Tapir	1	0	0	0
240	M	Picking	Tapir	1	0	0	0
241	M	Picking	Tapir	1	0	0	0
242	M	Picking	Tapir	1	0	0	0
243	F	Picking	Hornbill	1	0	0	0
244	F	Picking	Hornbill	1	0	0	0
245	F	Picking	Hornbill	1	0	0	0
246	F	Picking	Hornbill	1	0	0	0
247	M	Picking	Tapir (had medicine)	1	1	1	1
248	M	Picking	Tapir (had medicine)	1	1	0	0
249	M	Picking	Tapir (had medicine)	1	1	0	0
250	M	Picking	Tapir (had medicine)	1	1	0	0
251	M	Picking	Tapir (had medicine)	1	1	0	0
252	M	Picking	Tapir (had medicine)	1	1	0	0
253	M	Picking	Tapir (had medicine)	1	1	0	0
254	M	Picking	Tapir (had medicine)	1	1	0	0
255	F	Picking	Tapir (had medicine)	1	0	0	0
256	F	Picking	Tapir (had medicine)	1	1	0	0

257	M	Picking	Tapir (had medicine)	1	1	0	0
258	M	Picking	Tapir (had medicine)	1	1	0	0
259	M	Picking	Tapir (had medicine)	1	1	0	0
260	M	Picking	Tapir (had medicine)	1	1	0	0
261	F	Picking	Tapir	1	1	0	0
262	F	Picking	Tapir	1	0	0	0
263	Nymph	Picking	Tapir	10	0	0	0
264	F	Picking	Female barking deer	1	0	0	0
265	F	Picking	Female barking deer	1	0	0	0
266	Larvae	Dragging	Tapir (stall)	50	1	0	0
267	Larvae	Dragging	Tapir (stall)	17	1	0	0
268	Larvae	Dragging	Male eld's deer (stall)	50	1	0	0
269	Larvae	Dragging	Male eld's deer (stall)	50	1	0	0
270	Larvae	Dragging	Male eld's deer (stall)	50	1	0	0
271	Larvae	Dragging	Male eld's deer (stall)	50	1	0	0
272	Larvae	Dragging	Male eld's deer (stall)	50	1	0	0
273	Larvae	Dragging	Male eld's deer (stall)	50	1	0	0
274	Larvae	Dragging	Male eld's deer (stall)	50	1	0	0
275	Larvae	Dragging	Male eld's deer (stall)	50	1	0	0
276	Larvae	Dragging	Male eld's deer (stall)	50	1	0	0
277	Nymph	Dragging	Tapir (stall)	10	0	0	0
278	Nymph	Dragging	Tapir (stall)	10	0	0	0
279	Nymph	Dragging	Tapir (stall)	4	0	0	0
280	Nymph	Dragging	Male eld's deer (stall)	5	1	0	0
281	F	Picking	Tapir (Typhoon)	1	0	0	0
282	F	Picking	Tapir (Typhoon)	1	0	0	0
283	F	Picking	Tapir (Typhoon)	1	0	0	0
284	F	Picking	Tapir (Typhoon)	1	0	1	0
285	F	Picking	Tapir (Typhoon)	1	0	0	1

286	F	Picking	Tapir (Typhoon)	1	0	1	0
287	F	Picking	Tapir (Typhoon)	1	0	0	0
288	F	Picking	Tapir (Typhoon)	1	0	1	1
289	F	Picking	Tapir (Typhoon)	1	0	0	1
290	F	Picking	Tapir (Typhoon)	1	0	1	0
291	M	Picking	Tapir (Typhoon)	1	0	1	0
292	F	Picking	Tapir (Nawin)	1	0	1	0
293	F	Picking	Tapir (Nawin)	1	0	1	1
294	F	Picking	Tapir (Nawin)	1	0	1	1
295	F	Picking	Tapir (Nawin)	1	0	1	1
296	F	Picking	Tapir (Nawin)	1	1	0	0
297	F	Picking	Tapir (Nawin)	1	1	0	0
298	F	Picking	Tapir (Nawin)	1	1	0	0
299	F	Picking	Tapir (Nawin)	1	1	0	0
300	M	Picking	Tapir (Nawin)	1	1	0	0
301	M	Picking	Tapir (Nawin)	1	1	0	1
302	M	Picking	Tapir (Nawin)	1	1	1	1
303	M	Picking	Tapir (Nawin)	1	1	0	0
304	Nymph	Dragging	Tapir (stall)	5	1	0	0
305	Nymph	Dragging	Male eld's deer (stall)	1	1	0	0
306	Larvae	Dragging	Tapir (stall)	50	1	0	0
307	Larvae	Dragging	Tapir (stall)	50	1	0	0
308	Larvae	Dragging	Tapir (stall)	14	1	0	0
309	Larvae	Dragging	Male eld's deer (stall)	50	1	1	1
310	Larvae	Dragging	Male eld's deer (stall)	50	1	1	1
311	Larvae	Dragging	Male eld's deer (stall)	50	1	0	1
312	Larvae	Dragging	Male eld's deer (stall)	50	1	1	1
313	Larvae	Dragging	Male eld's deer (stall)	50	1	1	1
314	Larvae	Dragging	Male eld's deer (stall)	50	1	1	1

315	Larvae	Dragging	Male eld's deer (stall)	50	1	0	1
316	Larvae	Dragging	Male eld's deer (stall)	50	1	0	1
317	Larvae	Dragging	Male eld's deer (stall)	50	1	1	1
318	Larvae	Dragging	Male eld's deer (stall)	50	1	0	1
319	Larvae	Dragging	Male eld's deer (stall)	8	1	0	1
320	Larvae	Dragging	Male eld's deer (stall)	41	1	0	0
321	Larvae	Dragging	Tapir (stall)	47	1	0	0
322	Nymph	Dragging	Tapir (stall)	9	0	0	1
323	Nymph	Dragging	Male eld's deer (stall)	2	1	0	0
324	Larvae	Dragging	Male eld's deer (stall)	50	1	0	1
325	Larvae	Dragging	Male eld's deer (stall)	50	0	1	1
326	Larvae	Dragging	Male eld's deer (stall)	50	1	1	1
327	Larvae	Dragging	Male eld's deer (stall)	50	1	1	1
328	Larvae	Dragging	Male eld's deer (stall)	50	1	0	0
329	Larvae	Dragging	Male eld's deer (stall)	50	1	0	1
330	Larvae	Dragging	Male eld's deer (stall)	50	1	0	1
331	Larvae	Dragging	Male eld's deer (stall)	50	1	0	1
332	Larvae	Dragging	Male eld's deer (stall)	50	1	0	1
333	Larvae	Dragging	Male eld's deer (stall)	50	1	0	0
334	Larvae	Dragging	Male eld's deer (stall)	50	1	1	0
335	Larvae	Dragging	Male eld's deer (stall)	50	1	0	1
336	Larvae	Dragging	Male eld's deer (stall)	50	1	1	1
337	Larvae	Dragging	Male eld's deer (stall)	50	1	1	1
338	Larvae	Dragging	Male eld's deer (stall)	50	1	1	1
339	Larvae	Dragging	Male eld's deer (stall)	50	1	1	1
340	Larvae	Dragging	Male eld's deer (stall)	50	1		1
341	Larvae	Dragging	Male eld's deer (stall)	50	1	1	1
342	Larvae	Dragging	Male eld's deer (stall)	50	1	1	1
343	Larvae	Dragging	Male eld's deer (stall)	50	1	1	1

344	Larvae	Dragging	Male eld's deer (stall)	50	1	1	1
345	Larvae	Dragging	Male eld's deer (stall)	50	1	1	1
346	Larvae	Dragging	Male eld's deer (stall)	50	1	0	1
347	Larvae	Dragging	Male eld's deer (stall)	50	1	1	1
348	Larvae	Dragging	Male eld's deer (stall)	50	1	0	1
349	Larvae	Dragging	Male eld's deer (stall)	50	1	0	1
350	Larvae	Dragging	Male eld's deer (stall)	50	1	0	1
351	Larvae	Dragging	Male eld's deer (stall)	50	1	0	1
352	Larvae	Dragging	Male eld's deer (stall)	50	1	0	0
353	Larvae	Dragging	Male eld's deer (stall)	50	1	0	0
354	Larvae	Dragging	Male eld's deer (stall)	50	1	0	0
355	Larvae	Dragging	Male eld's deer (stall)	50	1	0	0
356	Larvae	Dragging	Male eld's deer (stall)	50	1	0	0
357	Larvae	Dragging	Male eld's deer (stall)	50	1	0	0
358	Larvae	Dragging	Male eld's deer (stall)	50	1	0	0
359	Larvae	Dragging	Male eld's deer (stall)	50	1	0	0
360	Larvae	Dragging	Male eld's deer (stall)	50	1	0	0
361	Larvae	Dragging	Male eld's deer (stall)	50	1	0	0
362	Larvae	Dragging	Male eld's deer (stall)	50	1	0	0
363	Larvae	Dragging	Male eld's deer (stall)	50	1	0	0
364	Larvae	Dragging	Male eld's deer (stall)	50	1	0	0
365	Larvae	Dragging	Male eld's deer (stall)	50	1	0	0
366	Larvae	Dragging	Male eld's deer (stall)	50	1	0	0
367	Larvae	Dragging	Male eld's deer (stall)	50	1	0	0
368	Larvae	Dragging	Male eld's deer (stall)	50	1	0	0
369	Larvae	Dragging	Male eld's deer (stall)	50	1	0	0
370	Larvae	Dragging	Male eld's deer (stall)	50	1	0	0
371	Larvae	Dragging	Male eld's deer (stall)	50	1	1	1
372	Larvae	Dragging	Male eld's deer (stall)	50	1	0	1

373	Larvae	Dragging	Male eld's deer (stall)	50	1	0	1
374	Larvae	Dragging	Male eld's deer (stall)	50	1	0	1
375	Larvae	Dragging	Male eld's deer (stall)	50	1	0	1
376	Larvae	Dragging	Male eld's deer (stall)	50	1	0	1
377	Larvae	Dragging	Male eld's deer (stall)	50	1	0	1
378	Larvae	Dragging	Male eld's deer (stall)	50	1	0	1
379	Larvae	Dragging	Male eld's deer (stall)	50	1	0	1
380	Larvae	Dragging	Male eld's deer (stall)	50	1	0	1
381	Larvae	Dragging	Male eld's deer (stall)	50	1	1	1
382	Larvae	Dragging	Male eld's deer (stall)	50	1	0	1
383	Larvae	Dragging	Male eld's deer (stall)	50	1	0	0
384	Larvae	Dragging	Male eld's deer (stall)	50	1	1	1
385	Larvae	Dragging	Male eld's deer (stall)	43	1	1	0
386	Larvae	Dragging	Male eld's deer (stall)	11	1	1	0
387	Nymph	Picking	Eld's deer K222	1	0	0	1
388	Nymph	Picking	Female spotted deer	4	0	0	1
389	Nymph	Picking	Eld's deer K294	10	0	0	0
390	Nymph	Picking	Eld's deer K222	1	0	1	0
391	Nymph	Picking	Hybrid cow	5	1	0	0
392	F	Picking	Spotted deer	1	1	0	0
393	M	Picking	Tapir (Kanya)	1	1	0	1
394	F	Picking	Hybrid cow	1	0	0	0
395	F	Picking	Hybrid cow	1	0	0	0
396	F	Picking	Hybrid cow	1	0	0	0
397	F	Picking	Eld's deer K295	1	0	0	0
398	F	Picking	Eld's deer K295	1	0	1	0
399	M	Picking	Eld's deer K295	1	0	0	1
400	F	Picking	Hybrid cow	1	0	0	0
401	F	Picking	Hybrid cow	1	0	0	0

402	F	Picking	Hybrid cow	1	0	0	0
403	F	Picking	Eld's deer K222	1	0	0	0
404	F	Picking	Eld's deer K222	1	0	0	0
405	M	Picking	Eld's deer K222	1	0	0	0
406	F	Picking	Eld's deer K205	1	0	0	0
407	F	Picking	Eld's deer K205	1	0	0	0
408	M	Picking	Hybrid cow	1	0	0	0
409	F	Picking	Hybrid cow	1	0	1	0
410	F	Picking	Hybrid cow	1	0	1	0
411	F	Picking	Hybrid cow	1	0	0	1
412	F	Picking	Hybrid cow	1	0	0	0
413	F	Picking	Hybrid cow	1	0	0	0
414	F	Picking	Hybrid cow	1	0	0	0
415	Larvae	Dragging	Tapir (stall)	50	1	0	1
416	Larvae	Dragging	Tapir (stall)	50	1	0	1
417	Larvae	Dragging	Tapir (stall)	50	1	0	0
418	Larvae	Dragging	Tapir (stall)	50	1	0	0
419	Larvae	Dragging	Tapir (stall)	50	1	0	0
420	Larvae	Dragging	Tapir (stall)	50	1	0	0
421	Larvae	Dragging	Tapir (stall)	50	1	0	0
422	Larvae	Dragging	Tapir (stall)	50	1	0	0
423	Larvae	Dragging	Tapir (stall)	50	0	0	0
424	Larvae	Dragging	Tapir (stall)	41	0	0	0
425	Larvae	Dragging	Tapir (stall)	50	1	0	0
426	Larvae	Dragging	Male eld's deer (stall)	50	0	0	0
427	Larvae	Dragging	Male eld's deer (stall)	50	0	0	0
428	Larvae	Dragging	Male eld's deer (stall)	50	0	0	0
429	Larvae	Dragging	Male eld's deer (stall)	50	0	0	0
430	Larvae	Dragging	Male eld's deer (stall)	50	1	0	0

431	Larvae	Dragging	Male eld's deer (stall)	50	1	0	0
432	Larvae	Dragging	Male eld's deer (stall)	50	1	0	0
433	Larvae	Dragging	Male eld's deer (stall)	50	0	0	0
434	Larvae	Dragging	Male eld's deer (stall)	50	0	0	0
435	Larvae	Dragging	Male eld's deer (stall)	50	0	0	0
436	Larvae	Dragging	Male eld's deer (stall)	50	0	0	0
437	Larvae	Dragging	Male eld's deer (stall)	50	0	0	0
438	Larvae	Dragging	Male eld's deer (stall)	50	0	0	0
439	Larvae	Dragging	Male eld's deer (stall)	50	0	0	0
440	Larvae	Dragging	Male eld's deer (stall)	50	0	0	0
441	Larvae	Dragging	Male eld's deer (stall)	50	0	0	0
442	Larvae	Dragging	Male eld's deer (stall)	50	0	0	0
443	Larvae	Dragging	Male eld's deer (stall)	50	0	0	0
444	Larvae	Dragging	Male eld's deer (stall)	50	0	0	0
445	Larvae	Dragging	Male eld's deer (stall)	50	0	0	0
446	Larvae	Dragging	Male eld's deer (stall)	30	0	0	0
447	Nymph	Dragging	Tapir (stall)	10	0	0	0
448	Nymph	Dragging	Tapir (stall)	5	0	0	0
449	Nymph	Dragging	Male eld's deer (stall)	2	0	0	0
449				10,436	248	73	133
